# A mechanistic quantitative systems pharmacology model platform for translational efficacy evaluation and checkpoint combination design of bispecific immuno-modulatory antibodies

**DOI:** 10.3389/fphar.2025.1571844

**Published:** 2025-04-10

**Authors:** Yiyang Xu, Siyuan Yang, Qi Rao, Yuan Gao, Guanyue Zhou, Dongmei Zhao, Xinsheng Shi, Yi Chai, Chen Zhao

**Affiliations:** ^1^ School of Pharmacy, Nanjing Medical University, Nanjing, China; ^2^ QSPMed Technologies, Nanjing, China; ^3^ Nanjing Sanhome Pharmaceutical Co., Ltd., Nanjing, China; ^4^ Phase I Clinical Trial Unit, The First Affiliated Hospital of Nanjing Medical University, Nanjing, China; ^5^ Department of Oncology, The First Affiliated Hospital of Nanjing Medical University, Nanjing, China

**Keywords:** quantitative systems pharmacology, immune checkpoint, bispecific antibody, therapeutic combination, model-informed drug development

## Abstract

Over the past 2 decades, tumor immunotherapies have witnessed remarkable advancements, especially with the emergence of immune checkpoint-targeting bispecific antibodies. However, a quantitative understanding of the dynamic cross-talking mechanisms underlying different immune checkpoints as well as the optimal dosing and target design of checkpoint-targeting bispecific antibodies still remain challenging to researchers. To address this challenge, we have here developed a multi-scale quantitative systems pharmacology (QSP) model platform that integrates a diverse array of immune checkpoints and their interactive functions. The model has been calibrated and validated against an extensive collection of multiscale experimental datasets covering 20+ different monoclonal and bispecific antibody treatments at over 60 administered dose levels. Based on high-throughput simulations, the QSP model platform comprehensively screened and characterized the potential efficacy of different bispecific antibody target combination designs, and model-based preclinical population-level simulations revealed target-specific dose-response relationships as well as alternative dosing strategies that can maintain anti-tumor treatment efficacy while reducing dosing frequencies. Model simulations also pointed out that combining checkpoint-targeting bispecific antibodies with monoclonal antibodies can lead to significantly enhanced anti-tumor efficacy. Our mechanistic QSP model can serve as an integrated precision medicine simulation platform to guide the translational research and clinical development of checkpoint-targeting immuno-modulatory bispecific antibodies.

## Introduction

T cells are now recognized as central players in the immune surveillance and cytotoxicity against tumors (Waldman et al., 2020). In the past decade, various classes of tumor immunotherapies harnessing T cell immunity, such as immune checkpoint inhibitors (ICIs), T cell engagers, and adoptive cellular therapies, have emerged as promising clinical treatment for cancer patients (Zhang and Zhang, 2020). Within the tumor microenvironment, different immune checkpoints (inhibitory and stimulatory) expressed on T cells, tumor cells and other immune cells dynamically interact with each other, and typically the inhibitory checkpoint functions would dominate and thereby drive evasion of immune detection and suppression of anti-tumor immune response (He and Xu, 2020). Therefore, the field of ICIs have attracted enormous research and made significant progress with a number of approved drugs in a wide range of cancer types. In 2011, Ipilimumab, a monoclonal antibody (mAb) targeting cytotoxic T-lymphocyte-associated protein 4 (CTLA4), became the first ICI approved by the U.S. Food and Drug Administration (FDA) for treating advanced melanoma (Mansh, 2011). Another checkpoint axis demonstrated to have a critical role in the regulation of T cells is programmed death receptor-1 (PD-1)/programmed cell death ligand 1 (PD-L1), and this has led to several approved blockbuster mAbs targeting the PD-1/PD-L1 pathway (e.g., Nivolumab, Pembrolizumab, Atezolizumab, Durvalumab) (Li et al., 2019; Wu, 2021). The advancements in ICIs have substantially transformed the therapeutic landscape of cancer treatment, with more and more research and clinical trials investigating ICI combinations in recent years (Lim et al., 2021). The promise and success of ICI combination in cancer treatment has also catalyzed the design and development of bispecific antibodies (BsAbs) that can simultaneous target two different immune checkpoints. By targeting two immuno-modulatory checkpoints and pathways (different from the mechanism of T cell or NK cell engagers), such BsAbs can potentially address the single-target limitations of classical mAbs to better boost immune cell activation and enhance anti-tumor efficacy. In 2022, Cadonilimab, an anti-PD-1/CTLA-4 bispecific antibody, has received regulatory approval in China for treating patients with relapsed or metastatic cervical cancer, marking the first ever approval of immuno-modulatory checkpoint-targeting bispecific antibody in the clinic (Keam, 2022). As interest in this area continues to grow, an increasing number of immuno-modulatory BsAbs targeting a long list of different immune checkpoints are progressing into clinical development, with more than 60 BsAbs in different clinical stages and many more in the preclinical research (Zhang et al., 2023; Klein et al., 2024).

The tumor microenvironment contains various types of innate and adaptive immune cells (as well as tumors cells) with differential expression of an array of inhibitory and stimulatory immune checkpoints, and it is now clear that their convoluted interactions can send dynamic cascaded signals via complex intracellular signaling to ultimately determine cell fate and immune activity (Anderson and Simon, 2020). For example, binding of PD-1 on T cells to PD-L1 expressed on tumor cells or antigen presenting cells (APCs) would first recruit protein tyrosine phosphatase-2 (SHP2) to dissociate proximal signaling molecules downstream of the T cell antigen receptor (TCR) and CD28 (Nagai, 2020a). This further dampens TCR-mediated signaling and downregulates key downstream proliferation-related pathways including the PI3K/AKT and MAPK axis (Bardhan et al., 2016; Wu et al., 2021). Similarly, T cell immunoglobulin and ITIM domains (TIGIT) on T cells can engage CD155 expressed on tumor cells and APCs to recruit SH2-containing inositol phosphatase-1 (SHIP1) to inhibit T cell signaling and activation (Liu et al., 2021). The checkpoint CTLA4 can shut down T cell activation by competitively inhibiting and blocking the CD28^−^CD80/86 co-stimulatory signal as it has higher affinity for CD80/CD86 compared to CD28 (Willsmore et al., 2021a; Alegre et al., 2001). Lymphocyte activation gene-3 (LAG3) can induce the sequestration of a key signaling kinase, lymphocyte-specific protein tyrosine kinase (Lck), from its co-receptor CD4/CD8, and this prevents the initiation of TCR-induced activation signals in T cells (Hivroz, 2022). On the other hand, stimulatory immune checkpoints such as tumor necrosis factor receptor superfamily members 4-1BB and OX40 are shown to play important roles in promoting T cell proliferation and activation (Webb et al., 2016; Martinez-Perez et al., 2021). Upon interaction with their respective ligands (4-1BBL and OX40L), these receptors recruit tumor necrosis factor-associated receptors (TRAFs) to transmit stimulatory signals and activate downstream hub proteins involving nuclear factor kappa B (NF-κB) and AKT, thereby enhancing T cell activation and cytokine production (Kim et al., 2022a; Sanchez-Paulete et al., 2016). As mentioned above, the different immune checkpoints can employ various mechanisms and intracellular signaling processes to collectively influence T cell function and anti-tumor cytotoxicity within the tumor microenvironment; furthermore, their dynamic binding and competition at the cell-surface level adds another layer of mechanistic complexity to this multi-cellular multi-scale system. As a result, such complexity can pose challenges to the research and development of new effective immuno-modulatory BsAbs and combination checkpoint-targeting treatments.

To address this, mechanism-based quantitative systems pharmacology (QSP) modeling has a great potential in helping us better understand this complex biosystem and guiding the optimal target selection as well as dosing design/evaluation of novel immuno-modulatory BsAbs. While a number of mechanism-based clinical-level QSP models (such as the QSP-IO platform and its series of modeling investigations) have been formulated to study patient treatment response and clinical dosing design in immuno-oncology (Sové et al., 2020; Milberg et al., 2019; Wang et al., 2023), only a few models have been developed specifically to guide the preclinical research and translation of BsAbs (including for immuno-modulatory and T cell engagers) and checkpoint-targeting mAbs (Kosinsky et al., 2018; Ma et al., 2021; Qiao et al., 2022). For example, Ma et al. formulated a mechanistic model for PBMC-humanized mouse that integrated T cell dynamics and tumor growth, and they utilized the model to assess mouse-specific response to T cell engagers in order to provide support for preclinical drug development (Ma et al., 2021). Qiao et al. constructed a preclinical QSP model to investigate the potential variability of anti-CTLA4 treatment in syngeneic mouse to derive biomarkers for human extrapolation (Qiao et al., 2022). Despite these advancements, prior modeling works are mostly cell-level models that rarely delved into checkpoint-mediated T cell intracellular signaling which critically controls tumor cytotoxicity as explained earlier. Moreover, published preclinical QSP models tend to focus on only one immune checkpoint and lack generalizability for therapies or new modalities (e.g., BsAb) targeting different checkpoint combinations. To address these limitations, we have here developed a new QSP model platform to guide the translational efficacy evaluation and target combination design of bispecific checkpoint-targeting immuno-modulatory antibodies. The model platform included more than 10 major immune checkpoints and was calibrated and validated using multiscale *in vitro/vivo* datasets obtained for over 20 BsAbs and mAbs evaluated at over 60 administered dose levels. Using the QSP platform, we comprehensively and comparatively assessed the efficacy of an array of bispecific antibody target combinations and revealed target-specific dose-response relationships to aid preclinical experimental design. The QSP model was also employed to evaluate the feasibility of alternative dosing strategies to maintain anti-tumor efficacy with reduced frequency, and simulations showed that the immuno-modulatory BsAb plus mAb combination treatment strategy can significantly enhance anti-tumor efficacy. Our mechanistic QSP model can serve as a high-throughput simulation platform to expedite translational research and clinical development of new immuno-modulatory bispecific antibodies and treatment combinations.

## Results

### Overview of the QSP model

**FIGURE 1:**
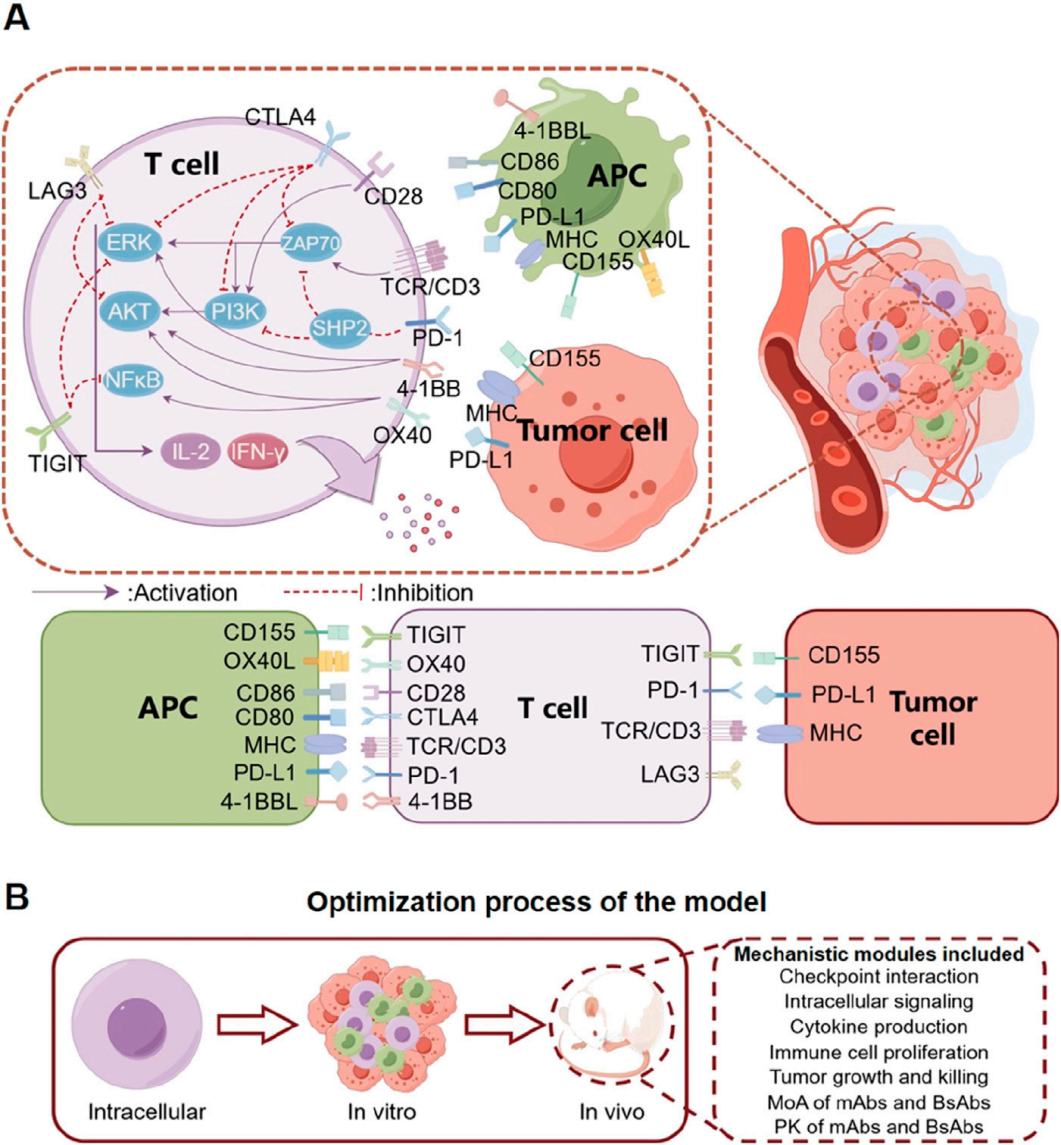
Diagram describing the mechanistic model structure including tumor cells, T cells and APCs. **(A)** The model primarily considers the interaction between T cells, APCs, and tumor cells within the tumor microenvironment, as well as the interaction between a number of immune checkpoints that can modulate T cell activation and anti-tumor cytotoxicity. Mono-specific (mAbs) and bispecific antibody (BsAbs) therapeutics can bind their corresponding immune checkpoints and regulate these processes to boost T cell response and tumor shrinkage. **(B)** We designed the model based on a pathway that starts from cell signaling pathways, proceeds to *in vitro* cell co-culture, and ultimately extends to *in vivo* studies in mice. The mouse model incorporates multiple modules, enabling it to meticulously elucidate the pharmacodynamics of diverse antibodies within mice.

The major components of our QSP model included the immune checkpoint-mediated interactions between T cells, tumor cells, and APCs, as well as *in vivo* drug pharmacokinetics and targeting. For the immune part, we focused on the TCR-mediated signaling pathway and major immune checkpoints including PD-1, PD-L1, CD28, CD80/CD86, LAG3, CTLA4, TIGIT, CD155, OX40, OX40L, 4-1BB, and 4-1BBL (expressed on T cells, tumor cells, or APCs) (Figure 1). In the model, T cell activation occurs through the binding of TCR/CD3 complex to major histocompatibility complex (MHC) on APCs and tumor cells, accompanied by the physical interaction between CD80/CD86 on APCs and CD28 on T cells (Boomer and Green, 2010; Bommhardt et al., 2019). For TCR-mediated cell signaling, we primarily included key intracellular signaling proteins and transcriptional factors (ZAP70, PI3K, NF-κB, ERK, and AKT) as well as their activating mechanisms (Hwang et al., 2020). Activated NF-κB, ERK, and AKT can mediate the release of IL-2 and IFN-γ from T cells, and these cytokines can in turn promote T cell proliferation and enhance tumor cell killing (Jorgovanovic et al., 2020; Setrerrahmane and Xu, 2017). On the other hand, the immune checkpoints interact correspondingly to exert their regulatory impact. Inhibitory immune checkpoints such as PD-1 binding to PD-L1 (on tumor cells and APCs) can recruit SHP2 and downregulate the phosphorylation of ZAP70 and PI3K in T cell intracellular signaling, thus driving T cells toward inactivation (Wu et al., 2021). Similarly, binding of TIGIT on T cells to CD155 on tumor cells and APCs can downregulate activation of ZAP70 and NF-κB in T cell intracellular signaling, which in turn inhibits T cell activation (Liu et al., 2022). The checkpoint LAG3 on T cells can induce the sequestration of key activating adaptor proteins from their co-receptors CD4/CD8, thereby preventing the initiation of TCR-mediated signaling as well as downstream signal transduction (Hivroz, 2022). Conversely, the stimulatory immune checkpoints can enhance T cell proliferation and function. In the model, binding between 4-1BB and 4-1BBL increases ERK and AKT phosphorylation in T cell intracellular signaling, while the OX40-OX40L binding between T cells and APCs can enhance the phosphorylation of AKT and NF-κB to drive T cell activation (Wang et al., 2009; Konstorum et al., 2019).

At the cell level, T cells can proliferate and mediate direct tumor cell killing. The mechanisms of different antibody therapeutics were included: they direct bind and sequester (if blocking antibody, e.g., PD-1) or activate (if activating antibody, e.g., 4-1BB) their checkpoint targets, thereby modulating checkpoint interaction and signaling (primarily on T cells). At the *in vivo* level, the biodistribution and pharmacokinetics of antibodies were included to estimate intratumoral drug concentration, and time-course tumor growth inhibition can be simulated by integrating all the aforementioned modules in the QSP model.

### Model-based characterization of T cell signaling and quantitative calibration using experimental data

**FIGURE 2:**
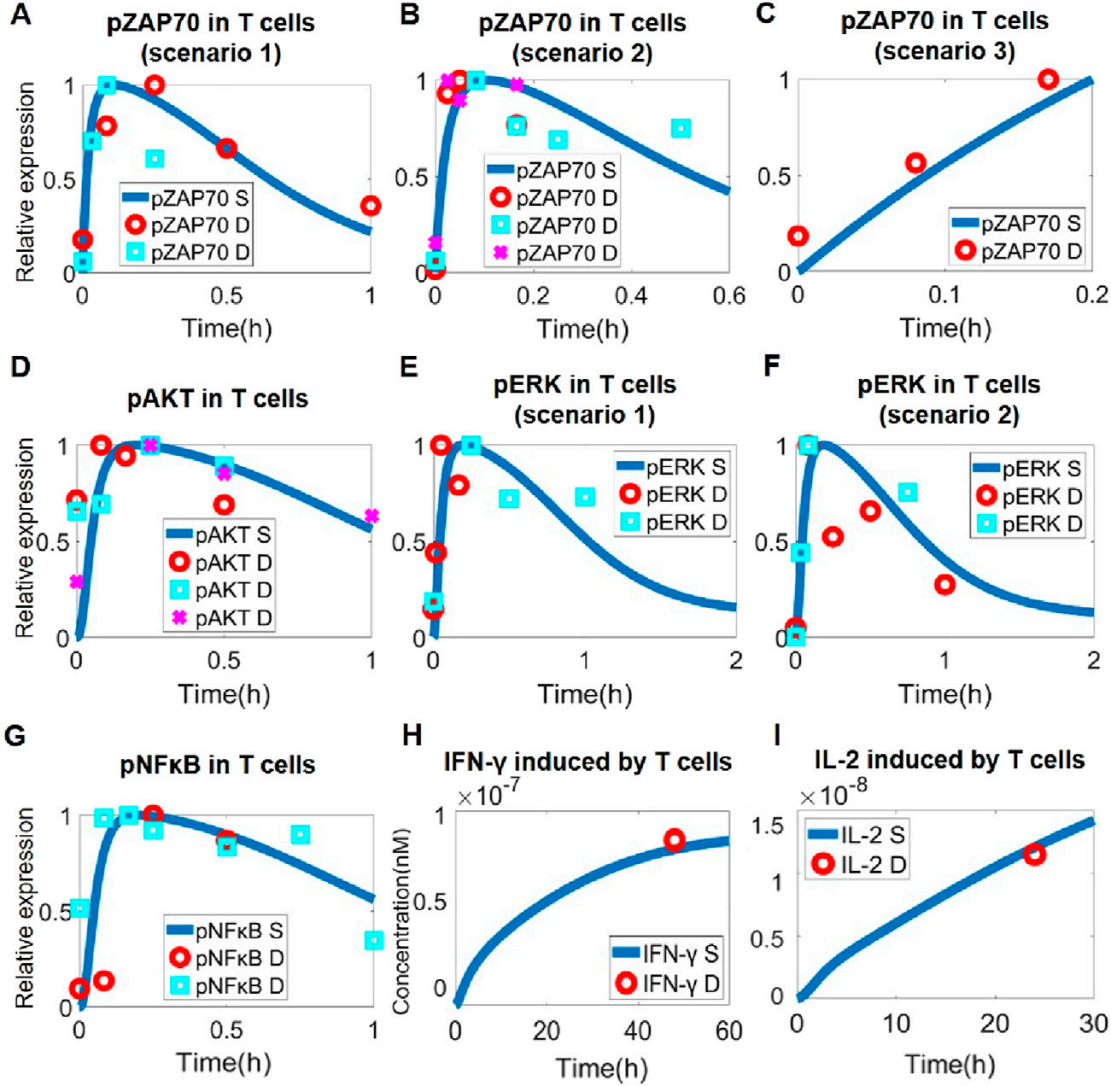
Model-based quantitative characterization of T cell intracellular signaling. Upon T cell activation, ZAP70 was rapidly phosphorylated, as shown by model simulation and experimental data in scenarios of **(A)** anti-CD3 treatment (data from and (Kästle et al., 2020; Tewari et al., 2021)), **(B)** anti-CD3 and anti-CD28 treatment (data from (Thumkeo et al., 2020) and (Cattley et al., 2020)), and **(C)** T-B cell co-culture (data from (Hui et al., 2017)). **(D)** Downstream activation of AKT by phosphorylation under anti-CD3 and anti-CD28 stimulation (data from (Narayan et al., 2006), (Zheng et al., 2019) and (Fu et al., 2021)). **(E)** Downstream activation of ERK by phosphorylation under anti-CD3 treatment (data from (Kassem et al., 2016) and (Lee et al., 2013)). **(F)** Downstream activation of pERK under anti-CD3 and anti-CD28 stimulation (data from (Kästle et al., 2020) and (Sheppard et al., 2004)). **(G)** In T cells, anti-CD3 and anti-CD28 stimulation activates NF-κB by phosphorylation (data from (Coudronniere et al., 2000) and (Clavijo and Frauwirth, 2012)). **(H)** Increased IFN-γ secretion by T cells after anti-CD3 stimulation (data from (Coto-Llerena et al., 2021)). **(I)** Increased IFN-γ secretion by T cells after anti-CD3 and anti-CD28 stimulation (data from (Wang et al., 2007)). **(A–G)** Y-axes are relative expression levels (normalized to their respective maximum values). S, simulation; D, experimental data.

In physiology, T cell activation happens primarily through TCR signaling and involves an array of downstream signaling proteins and axes. As the immune checkpoints are also known to regulate the phosphorylation of major signaling hub proteins like ZAP70, ERK, PI3K, AKT, and NF-κB (Wu et al., 2021; Hivroz, 2022; Nagai, 2020b; Kim et al., 2022b; Willsmore et al., 2021b; Liu et al., 2013), we therefore focused on these key proteins when formulating the intracellular signaling pathway that characterizes T cell activation in the QSP model. Upon activation of TCR and CD28, transient phosphorylation of ZAP70 and PI3K/AKT would first occur and lead to downstream cascaded activation of ERK and NF-κB (Figures 2A–G), which can promote the production and secretion of IL-2 and IFN-γ by T cells to enhance anti-tumor cytotoxicity (Figures 2H, I). To calibrate our model simulations, various experimental time-course protein phosphorylation and cytokine release datasets were employed (including T cell activation by antibody addition such as anti-CD3 and anti-CD28, as well as by APC co-culture). Overall, these quantitative data on T cell intracellular signaling were well captured by our QSP model (Figures 2A–I; Supplementary Figure S1).

### Integrative stepwise calibration and validation of the QSP model using *in vitro* and *in vivo* drug perturbation data

**FIGURE 3:**
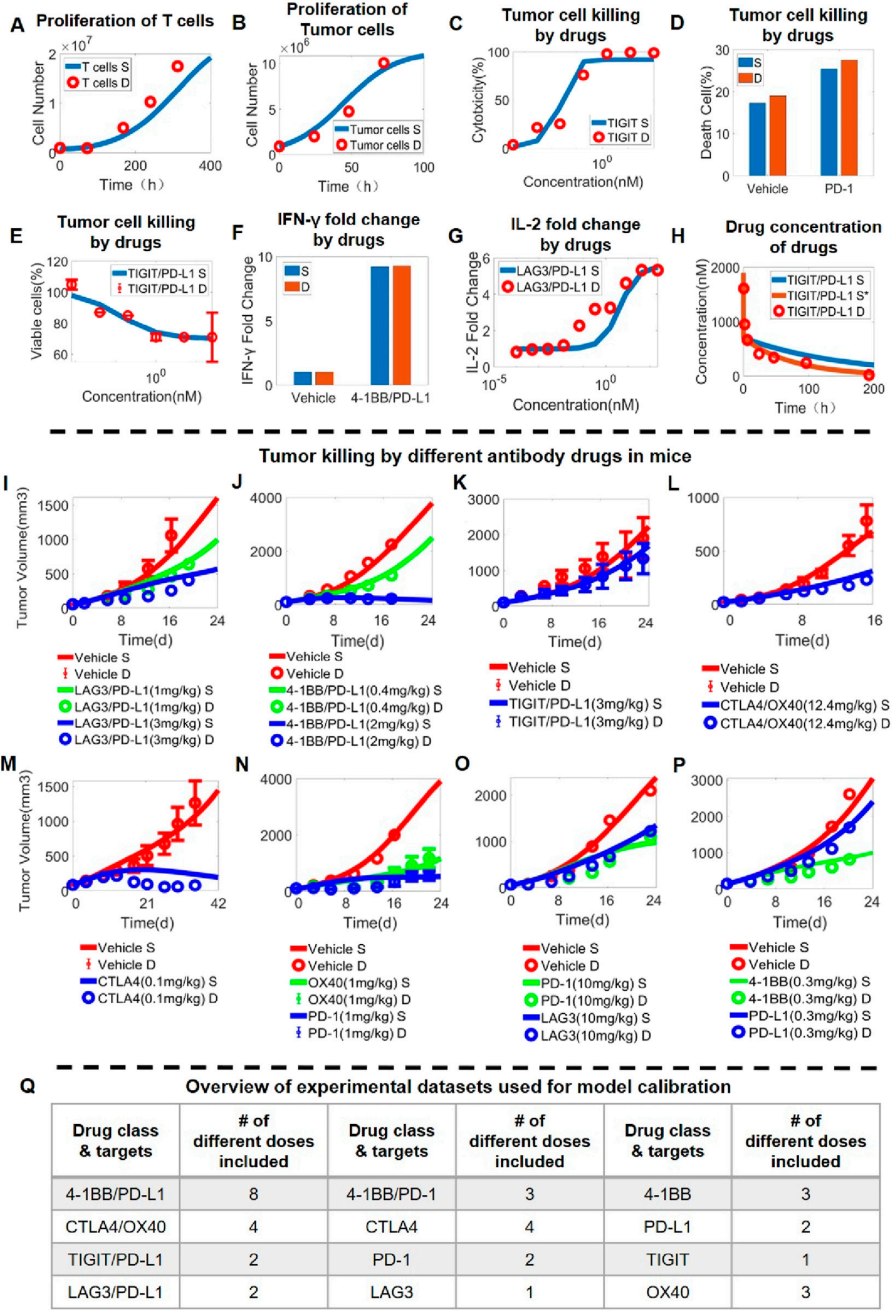
Stepwise model calibration using *in vitro* and *in vivo* data on antibody-induced tumor growth inhibition. The QSP model can quantitatively capture **(A)** the time-dependent proliferation of T cells (data from (Teschner et al., 2011)) and **(B)** the growth of tumor cells over time (data from (Khazen et al., 2019)). The integrated *in vitro* QSP model captures the dose response relationship of **(C)** anti-tumor cytotoxicity of anti-TIGIT antibody (data from (Zhong et al., 2022)), **(D)** anti-tumor cytotoxicity of anti-PD-1 antibody (data from (Wang et al., 2021)), **(E)** anti-tumor cytotoxicity of anti-TIGIT/PD-L1 bispecific antibody (data from (Yang et al., 2023)), as well as increase in **(F)** IFN-γ release after anti-4-1BB/PD-L1 bispecific antibody treatment (data from (Muik et al., 2022)) and **(G)** IL-2 release after anti-LAG3/PD-L1 bispecific antibody treatment (data from (Jiang et al., 2021)). **(H)** Plasma pharmacokinetics of anti-TIGIT/PD-L1 bispecific antibody in mice (data from (Mu et al., 2022)). **(I-P)** *In vivo* antitumor activity of different antibody treatment regimens targeting immune checkpoints (and administered at different doses) as characterized by the QSP model; examples shown here include **(I)** anti-LAG3/PD-L1 bispecific antibody (data from (Kraman et al., 2020)), **(J)** anti-4-1BB/PD-L1 bispecific antibody (data from Jeong et al., 2021), **(K)** anti-TIGIT/PD-L1 bispecific antibody (data form (Xiao et al., 2021)), **(L)** anti-CTLA4/OX40 bispecific antibody (data form (Kvarnhammar et al., 2019)), **(M)** anti-CTLA4 antibody (data from (Gan et al., 2022)), **(N)** anti-OX40 and anti-PD-1 antibodies (data from (Kuang et al., 2020)), **(O)** anti-LAG3 and anti-PD-1 antibodies (data from (Yu et al., 2019)), and **(P)** anti-4-1BB and anti-PD-L1 antibodies (data from (Cheng et al., 2022)). **(Q)** An overview table summarizing the complete *in vivo* experimental datasets used for model calibration. S, simulation; S*, second simulation after calibration; D, experimental data.

After we calibrated the T cell intracellular signaling module, we then integrated it into the QSP model at the *in vitro* level that considers checkpoint-mediated cell-cell interaction, proliferation and cytotoxicity. First, the behaviors of individual cell components (T cell, tumor cell) were optimized so that the experimentally observed cell growth kinetics can be reproduced by the model simulations (Figures 3A, B; Supplementary Figure S2A, B). Then, in T-tumor co-culture scenarios, the *in vitro* QSP model can quantitatively and simultaneously predict the tumor cell killing dose response upon antibody treatments (mAbs and BsAbs) targeting different immune checkpoints such as TIGIT (Figure 3C), PD-1 (Figure 3D), PD-L1 (Supplementary Figure S2) and TIGIT/PD-L1 (Figure 3E; Supplementary Figure S2). In addition to cytotoxicity, our QSP model was able to characterize the increase in cytokine release in T-APC co-culture scenarios upon immune-modulatory antibody treatment (covering an array of checkpoint targets such as PD-L1, 4-1BB, LAG3, CTLA4 and considering both mAbs and BsAbs) (Figures 3F, G; Figure 2E–G).

To further extend the model framework to *in vivo* scenarios, pharmacokinetic (PK) modules for immune-modulatory antibodies were then added and calibrated against time-course *in vivo* mouse PK data. However, as mouse PK data were not readily available for all the different antibodies, we assumed that the same set of parameters can be derived for a general prediction of *in vivo* antibody mouse PK, and the validity of this assumption was supported through quantitatively benchmarking model prediction against a set of published *in vivo* PK data obtained for mAbs and BsAbs (Figures 3A–H). After the addition of antibody PK, we again calibrated the model parameters using quantitative *in vivo* tumor growth inhibition data (primarily in MC38 syngeneic mice) that were obtained for numerous immuno-modulatory mAbs and BsAbs (Figures 3I–P; Supplementary Figure S4, 5). Overall, the dataset used for *in vivo* QSP model calibration encompassed 12 different mAbs and BsAbs treatment regimens (covering all major immune checkpoints) with 35 doses tested and over 300 time-course datapoints included (Figure 3Q).

**FIGURE 4:**
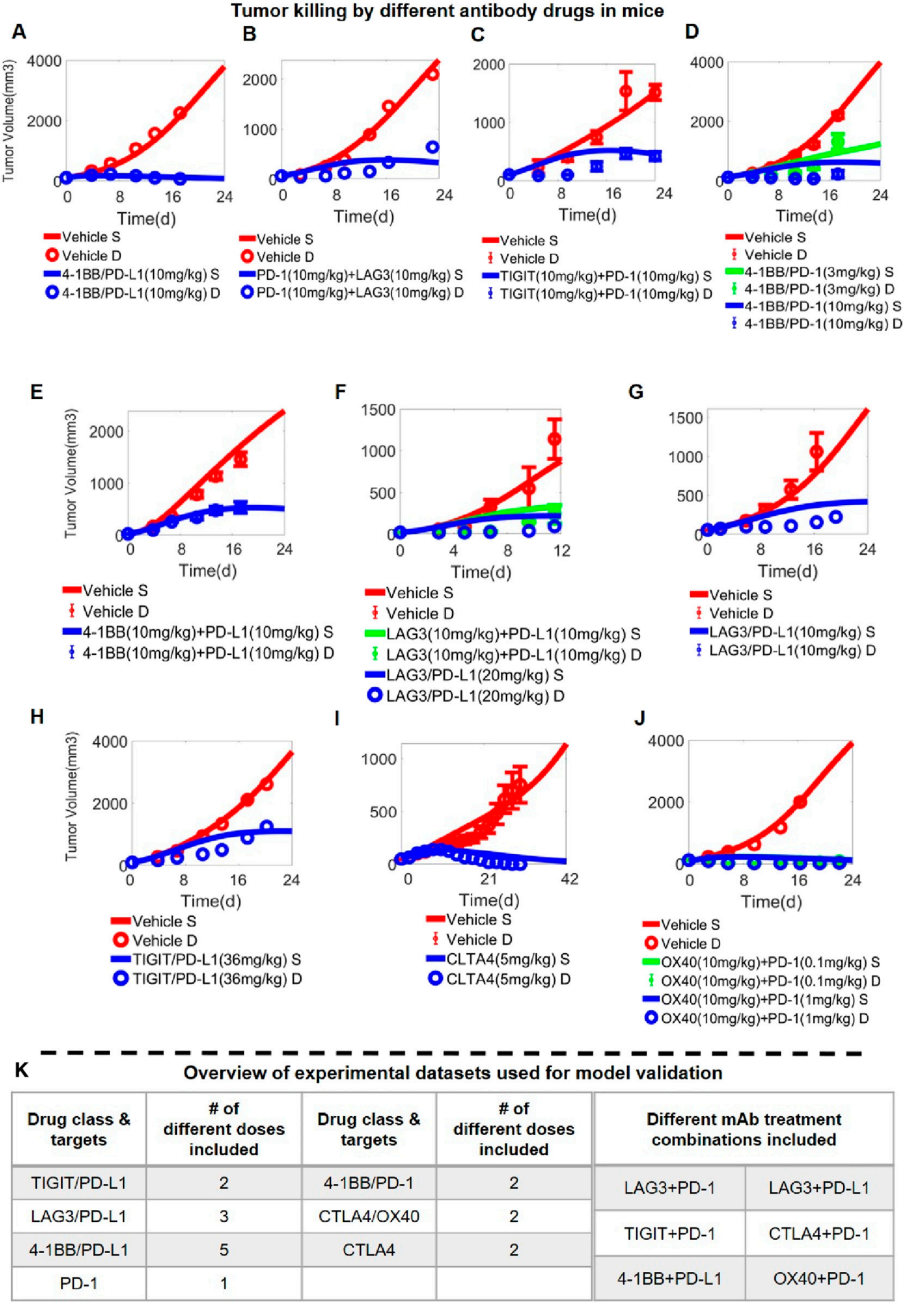
Model Validation using *in vivo* data on antibody-induced tumor growth inhibition. *In vivo* antitumor activity of different antibody treatment regimens targeting immune checkpoints (and administered at different doses) as predicted by the QSP model; examples shown here include **(A)** anti-4-1BB/PD-L1 bispecific antibody (data from (Jeong et al., 2021)), **(B)** anti-LAG3 and anti- PD-1 antibodies (data from (Yu et al., 2019)), **(C)** anti-TIGIT and anti- PD-1 antibodies (data from (Shao et al., 2021)), **(D)** anti-4-1BB/PD-1 bispecific antibody (data from (Qiao et al., 2021)), **(E)** anti-4-1BB and anti-PD-L1 antibodies (data from (Cheng et al., 2022)), **(F–G)** anti-LAG3/PD-L1 bispecific antibody (data from (Kraman et al., 2020)), **(F)** anti-LAG3 and anti-PD-L1 antibodies, **(H)** anti-TIGIT/PD-L1 bispecific antibody (data from (Zhong et al., 2022)), **(I)** anti-CTLA4 antibody (data from (Du et al., 2018)), **(J)** anti-OX40 and anti- PD-1 antibodies (data from (Kuang et al., 2020)). **(K)** A table summarizing the complete *in vivo* experimental datasets used for model validation. S, simulation; D, experimental data.

After the integrative QSP model was calibrated in stepwise manners using both *in vitro* and *in vivo* drug perturbation data, we aim to validate the model’s predictive capacity using new *in vivo* data unused during model calibration. For that purpose, we reserved all the high dose treatment arms for the different immuno-modulatory mAbs and BsAbs in the mice experiments as the validation set and simultaneously compared such data with the corresponding model simulations (Figures 4A–J; Supplementary Figure S6), which included a total of 23 doses/regimens of antibody treatment (covering 5 BsAbs, two mAbs, and six different mAb combinations) (Figure 4K). Overall, the quantitative model simulations under different antibody treatment scenarios agree well with the reported experimental data (Figure 4; Supplementary Figure S6), indicating that our integrative QSP model has significant potential in characterizing and predicting the preclinical *in vivo* efficacy of new combination strategies targeting these immune checkpoints.

### Model-based global sensitivity analysis and anti-tumor efficacy prediction of BsAbs with different checkpoint targets

**FIGURE 5:**
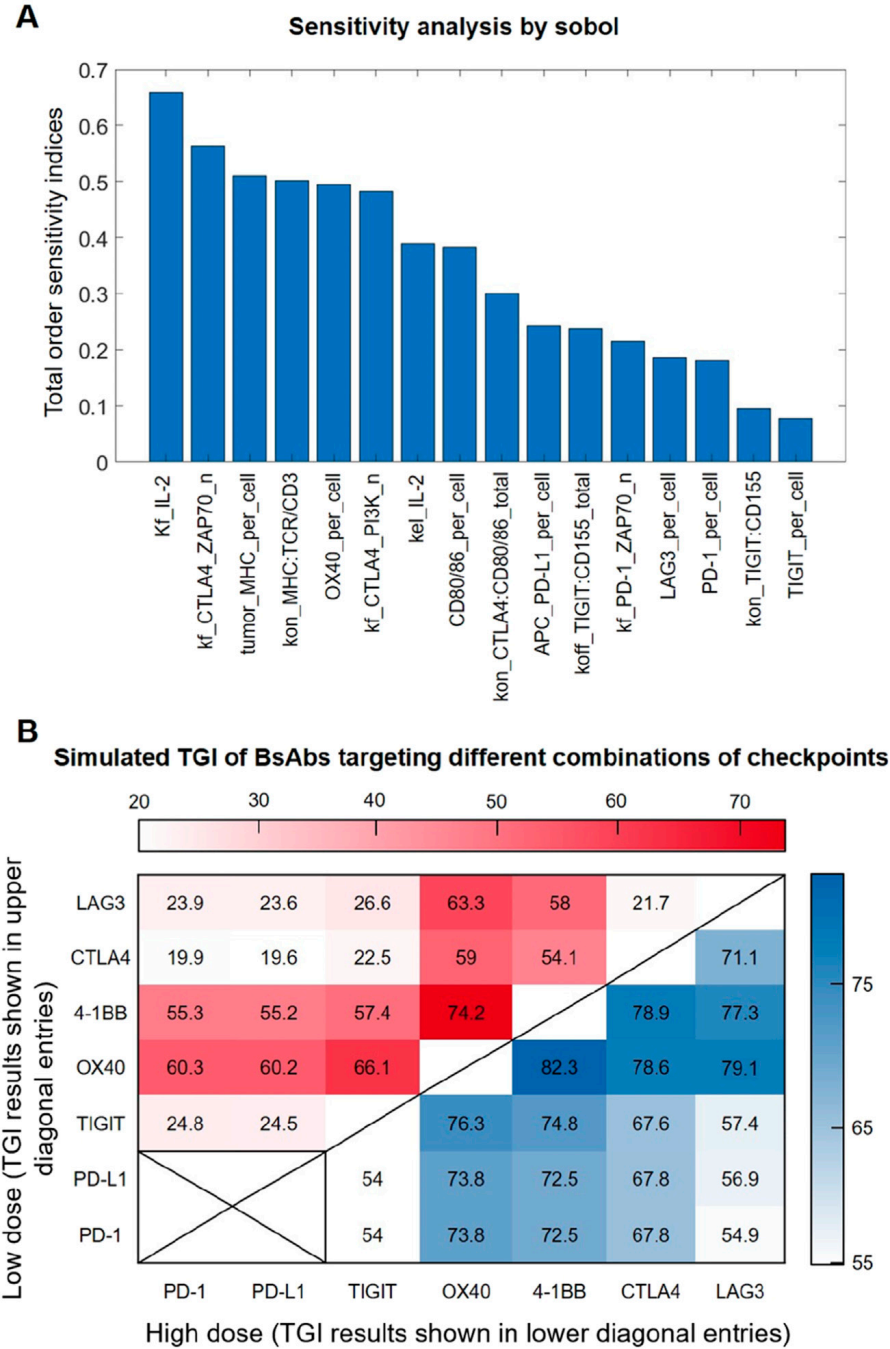
Sensitivity analysis and simulation of anti-tumor efficacy of BsAbs with different checkpoint targets. **(A)** The Sobol indices of the top-ranked parameters predicted have a significant impact on tumor growth. **(B)** Model-predicted *in vivo* tumor growth inhibition (TGI) values under different BsAb treatment that targets different combinations of immune checkpoints. Each (X,Y) entry represents a potential BsAb drug that simultaneously targets X and Y checkpoints, with its simulated TGI results under the low dose (1 mg/kg) and high dose (10 mg/kg) conditions shown in the upper diagonal region (in red heatmap) and lower diagonal region (in blue heatmap) respectively.

We conducted global sensitivity analysis on the *in vivo* level QSP model using the Sobol method to identify the most influential parameters on the model output of interest, specifically tumor volume. Among the top-ranked parameters (besides the rate of tumor proliferation and death), the rate of IL-2 production (kf_IL-2) stood out as a crucial factor influencing the model output (Figure 5A), as IL-2 in the model can positively feedback to regulate T cell signaling and control T cell proliferation and thereby limiting tumor growth. In addition, parameters associated with immune checkpoint-mediated processes such as the rate of ZAP70 activation or inhibition by upstream checkpoint complexes also showed substantial impact (e.g., kf_CTLA4_ ZAP70_n, kf_PD-1_ZAP70_n). Besides, cell surface expression of certain immune checkpoints (e.g., OX40_per_cell, PD-1_per_cell, TIGIT_per_cell) were also suggested to be influential in terms of regulating tumor growth, as expected.

As drug development efforts are now looking into different combinations of immune checkpoints when designing bispecific antibodies, we therefore comprehensively analyzed all possible bispecific combinations of the seven major immune checkpoints included in our QSP model. The tumor growth inhibition (TGI) value *in vivo* (e.g., in MC38 mouse model) of these virtual BsAbs were predicted under low (1 mg/kg) and high doses (10 mg/kg), respectively. Simulation results indicate that different combinations of checkpoints employed by the BsAbs can result in highly different anti-tumor efficacy, although this efficacy difference tend to be smaller at higher treatment doses (Figure 5B). Simulations also suggested that the checkpoints 4-1BB and OX40 may be most influential when designing immune-modulatory BsAbs, possibly due to their immuno-activating functions, as BsAbs that include either of these two checkpoints as one activating arm were always predicted to have superior anti-tumor efficacy (Figure 5B).

### Model-based evaluation of dosing regimens for immuno-modulatory antibody therapeutics in preclinical research

**FIGURE 6:**
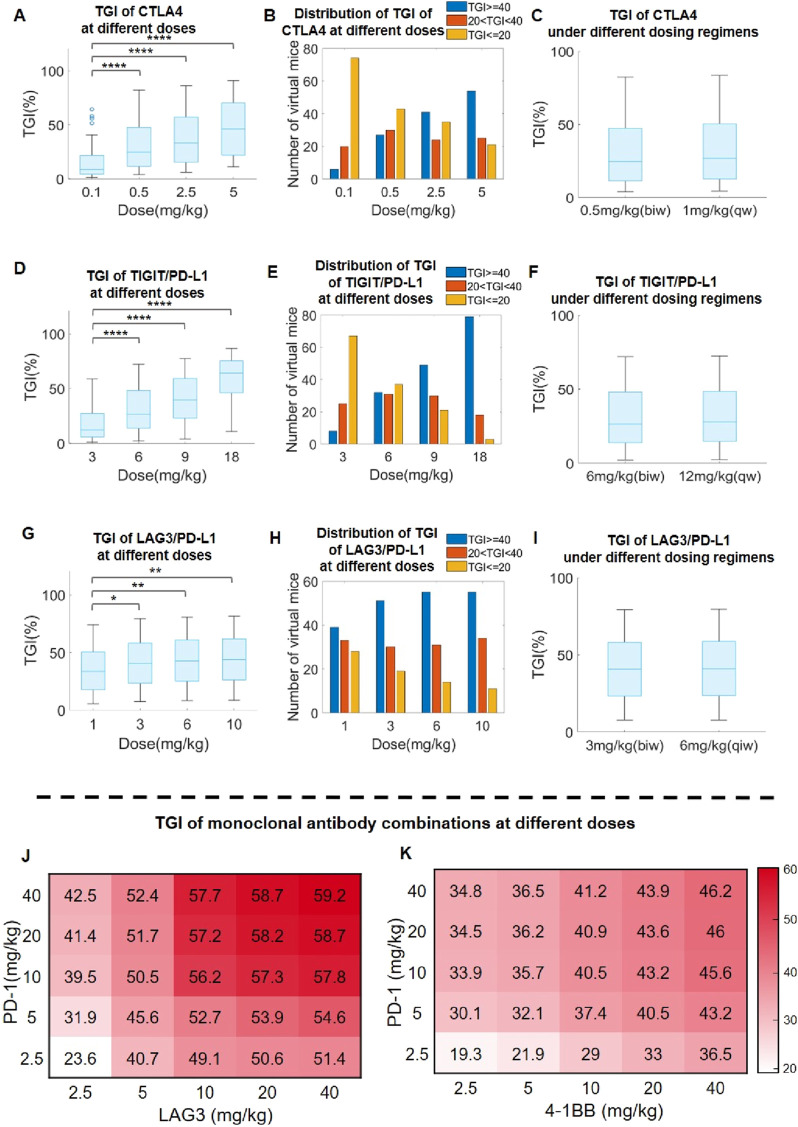
Model-based optimization of preclinical antibody dosing design in immuno-oncology research. **(A)** Predicted TGI in the virtual mouse population after anti-CTLA4 treatment at doses of 0.1–5 mg/kg, and **(B)** distribution of TGI response depth (percentages of mice with TGI ≥ 40%, 20%<TGI<40%, and TGI ≤ 20%) at different doses. **(C)** Predicted TGI in response to anti-CTLA4 treatment at 0.5 mg/kg biw (twice per week) and 1 mg/kg qw (once per week) dosing regimens. **(D)** Predicted TGI in the virtual mouse population after anti-TIGTI/PD-L1 BsAb treatment at doses of 3–18 mg/kg, and **(E)** distribution of TGI response depth at different doses. **(F)** Predicted TGI in response to anti-TIGTI/PD-L1 BsAb treatment at 6 mg/kg biw and 12 mg/kg qw regimens. **(G)** Predicted TGI in the virtual mouse population after anti-LAG3/PD-L1 BsAb treatment at doses of 1–10 mg/kg, and **(H)** distribution of TGI response depth at different doses. **(I)** Predicted TGI in response to anti-LAG3/PD-L1 BsAb treatment at 3 mg/kg biw and 6 mg/kg qw regimens. **(J–K)** Predicted population-level TGI heatmap for the combination of **(J)** anti-PD-1 and anit-LAG3, as well as **(K)** anti-PD-1 and anit-4-1BB, at different doses. ^∗^P < 0.05, ^∗∗^P < 0.01, ^∗∗∗∗^P < 0.0001. Statistical analyses were performed using Wilcoxon rank-sum test.

To evaluate the treatment dose response of immuno-modulatory antibody therapeutics in preclinical research settings, we created a virtual mouse population based on our QSP model (see Materials and Methods for details). Then, we simulated the population-level anti-tumor response after administering varying doses of different antibody therapeutics to the virtual mouse population. For anti-CTLA4 antibodies as an example mAb, simulations suggested a notable increase in overall population-level TGI as well as depth of response (e.g., percent of mice with large TGI values) along with increasing doses (Figures 6A, B). Different dosing regimens were also simulated (0.5 mg/kg twice per week *versus* 1 mg/kg once per week), and the results suggested comparable anti-tumor efficacy in mice (Figure 6C). For anti-TIGIT/PD-L1 antibodies as an example BsAb, model simulations also suggested a consistent increase in population-level TGI as well as depth of response with increasing doses (Figures 6C, D), as well as comparable efficacy between twice per week (halved dose) *versus* once per week (full dose) regimens (Figure 6F). However, the above trends were not universal for all immuno-modulatory antibodies. For example, in the case of anti-LAG3/PD-L1 BsAb, our model simulations predicted a near saturation effect at the 3 mg/kg dose, with minimal efficacy gain at higher doses (Figures 6G–I). We also evaluated some other example BsAbs and the results (displaying different dose response trends) were shown in Supplementary Figure S7.

We further investigated the utility of our QSP model in assessing optimal immuno-modulatory mAb combination dosing design. As the standard dosages used for co-administering two immuno-modulatory mAbs was typically around 10 mg/kg based on published literature, we therefore assessed the landscape of *in vivo* TGI in the virtual mouse population in response to combination mAb treatments at dosing ranges of 2.5–40 mg/kg. Using anti-PD-1 and anti-LAG3 combination as one example, we noted a saturation effect at 10 mg/kg for each mAb with marginal population-level TGI increase beyond this combination dose level (Figure 6J). Interestingly, in another example (anti-4-1BB and anti-PD-1 combination), the predicted trend is different and that the population-level TGI continues to rise along with increasing doses of anti-4-1BB but not that of anti-PD-1 (Figure 6K). In a similar way, we also explored the potential combination of one immune checkpoint mAb with SHP2 inhibition (a targeted mechanism with increasing significance in cancer treatment), and the results also suggested better efficacy in the combination scenario at the preclinical level (Supplementary Figure S8). The above results indicated the need for case-by-case dose-response evaluation and the potential utility of high-throughput QSP modeling in developing different immuno-modulatory combination strategies.

### QSP model-based exploration of triple checkpoint combination strategy

**FIGURE 7:**
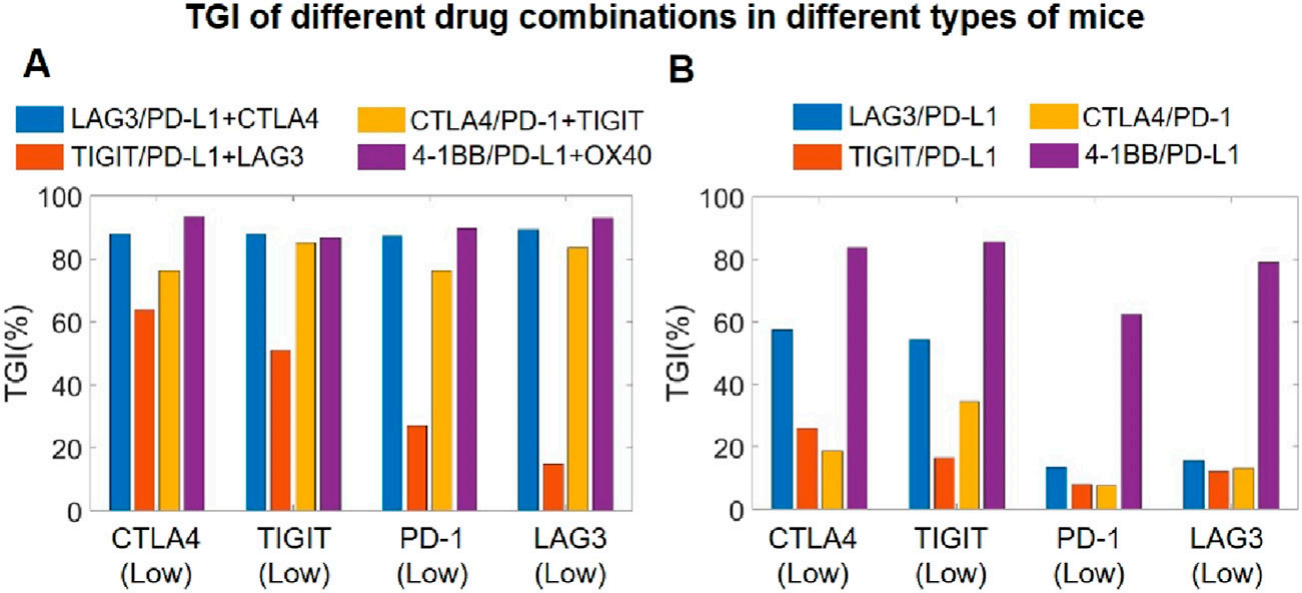
Model-based investigation of triple checkpoint combination strategies. **(A)** Predicted TGI in different virtual mouse populations (with differential checkpoint expression profiles) after mAb plus and BsAb combination treatment targeting three immune checkpoints, and comparisons with **(B)** predicted TGI in the same virtual mouse populations after only BsAbs treatment targeting two immune checkpoints. CTLA4 (Low), mice with low T cell expression of CTLA4; TIGTI (Low), mice with low T cell expression of TIGIT; PD-1 (Low), mice with low T cell expression of PD-1; LAG3 (Low), mice with low T cell expression of LAG3.

We also used our model to explore the potential efficacy of simultaneously targeting three immune checkpoints using antibody therapeutics, as this idea has attracted accumulating interest in the pharmaceutical community with pioneering companies already published encouraging data indicating early signals of superiority compared to standard single-agent BsAbs targeting two checkpoints (Huang et al., 2022). Addressing this topic of interest, we further created subgroups of virtual mouse populations, each exhibiting different immune checkpoint expression profiles in order to explore potential treatment efficacy in different tumor phenotypes. For the four different combination strategies tested, our QSP model simulations of the BsAb plus mAb combination (targeting three immune checkpoints simultaneously) suggested overall notable increase in TGI compared to single agent BsAb (Figures 7A, B). Among them, the triple combination of TIGIT/PD-L1/LAG3 appears to be less potent compared to the others, particularly in PD-1 low or LAG3 low tumors, whereas the 4-1BB/PD-L1/OX40 combination can yield the best TGI response across all types of tumors in mice (Figure 7A).

## Discussion

We have here developed a novel comprehensive QSP platform model for the translational evaluation of complex immune-modulatory antibody therapeutics. Our QSP model integrates T cell intracellular signaling, T cell and tumor cell proliferation, multi-cellular immune checkpoint interaction, mechanism of action of mAbs and BsAbs, and through extensive calibration and validation using multi-scale data, the model can accurately describe the preclinical efficacy of BsAbs targeting different immune checkpoints and assess optimal dosing regimens as well as combinations in tumor-bearing mice. Although the current model version includes only three major cell types (T cells, tumor cells, and APCs) in the tumor microenvironment, the future plan is to continue enriching the model to incorporate more immune cell types, for example, natural killer (NK) cells and CD4 and CD8 T cell subsets, as this can greatly enhance the model’s capability to capture the full complexity of the tumor-immune interactions. Additionally, the model currently assumed that the majority of immune checkpoints are expressed at constant levels on all cells (with the exception of CTLA4). Studies have revealed that checkpoints such as PD-L1 expressed on tumor cells can be induced by cytokine IFN-γ (Abiko et al., 2015), and thus characterizing the dynamic changes in the expression of different immune checkpoints on different cells is also a feature to be added in future model iterations. Regarding antibody PK, as the current model version utilized a calibrated typical PK profile for all antibody therapeutics due to limitation of data, future investigations should plug in experimentally-measured PK data specific to the antibody of interest (if available) to obtain more precise efficacy simulations.

The clinical limitations of mAbs in treating cancer patients, including drug resistance and low response rates, have spurred extensive research into BsAbs in recent years (Fan et al., 2015). Numerous studies have shown that BsAbs in immuno-oncology can effectively complement the intrinsic disadvantage of mAbs (targeting only one checkpoint), with dozens of BsAbs now in clinical trials. In theory, the more targets an antibody therapeutic can bind to, the higher its potential therapeutic effectiveness tends to be in modulating immune activation against cancer. Therefore, this field has also evolve beyond the traditional two-target BsAb approach, with researchers now actively exploring tri-specific and multi-specific immuno-modulatory antibodies as well as combination therapies that aim to regulate more than two checkpoints (Runcie et al., 2018). Following this idea, we have conducted extensive *in silico* efficacy analyses on the various different combination of an immuno-modulatory BsAbs with a mAb that together target three checkpoints at the same time. Our findings revealed that such combinations (of a BsAb with a mAb) can generally exhibit significantly higher potency compared to the BsAb alone in terms of higher TGI in tumor-bearing mice (Figure 7). Consistent with our findings, emerging experimental studies have also shown that immuno-modulatory antibody combinations targeting three checkpoints, e.g., anti-CTLA4/PD-L1 (BsAb) with anti-TIGIT (mAb) in Huang et al. or the tri-specific anti-PD-L1/TIGIT/LAG3 therapeutic in Yang et al., can deliver more pronounced T cell activation and anti-tumor potency compared to targeting only two checkpoints (e.g., by a BsAb) (Yang et al., 2023; Huang et al., 2022). However, there is chance that excessive immune checkpoint targeting could trigger severe inflammatory storms as side effects in patients. Moving forward, it is worthy to integrate an adverse effect module into the QSP model platform to detail the potential adverse impact of certain immune cells and inflammatory cytokines on normal tissue.

Another critical point that can be addressed by our QSP modeling strategy is the optimization of antibody dosing regimens, considering that the frequency of drug administration can influence patient compliance and quality of life. Through model simulations presented in the Results section, we observed that increasing the dosage while decreasing the frequency of BsAb administration can give rise to similar preclinical anti-tumor efficacy (Figure 6). Although the potential of such alternative dosing strategies has not yet been tested in immuno-modulatory BsAbs that target two checkpoints, clinical studies have shown that for immuno-modulatory mAbs targeting PD1/PD-L1 (e.g., nivolumab, pembrolizumab, durvalumab), dosing them at lower frequency and higher doses can result in comparable efficacy and safety profiles (Hosseini et al., 2020; Zhao et al., 2022). Therefore, it can be reasonably envisioned that immuno-modulatory BsAbs may also implement such alternative dosing strategies with the help of modeling and simulation in the near future. Overall, given the rising interest in developing effective BsAbs within the pharmaceutical industry (Klein et al., 2024), challenges remain in the successful clinical translation of such immuno-modulatory checkpoint-targeting BsAbs, and mechanism-based QSP modeling can be a critical tool in guiding decision-making throughout the entire preclinical-to-clinical development phases. Selecting the optimal combination of checkpoint targets is of crucial importance in BsAb design, as immune checkpoints may have widespread yet differential cell surface expression and sometimes unique or redundant functions. In this sense, a modeling platform like the one we developed can provide quantitative and explainable projections of BsAb’s anti-tumor efficacy to guide target selection and affinity optimization. Then, during first-in-human dose selection and dosing regimen design, mechanistic QSP modeling (integrating preclinical and clinical data) can provide valuable insights by prospectively simulating possible dosing scenarios in high-throughput manners and comparatively calculating potential risk-benefit profiles for patients. Efforts implementing such model-informed decision-making strategies have been increasingly recognized in the research and translation of innovative new drugs across different therapeutic areas (Hosseini et al., 2020; Zhao et al., 2022; Zhou et al., 2024). Still, it should be noted that the presented QSP platform primarily considered certain syngeneic mouse models as its main data source, and therefore more experimental data (from diverse preclinical animal models for immuno-oncology) and model-based analyses will always be needed to better fill the translational gap between mouse and human. Our future plan also includes extending the presented QSP model to the patient level and create diverse virtual patients to accurately simulate clinical population-level response of immune-modulatory antibody therapeutics to speed up bench-to-bedside translation.

## Materials and methods

### Summary of QSP model formulation

The presented QSP model is comprised of modules of T cell intracellular signaling, cell proliferation, T cell-tumor cell-APC checkpoint interaction, antibody *in vivo* pharmacokinetics, and preclinical anti-tumor cytotoxicity by T cells. The mechanisms of action of the different antibody drugs (mAbs and BsAbs) were implemented by referencing the mass-action binding kinetics as described in the related works by Betts et al. and Song et al. (Song et al., 2021; Betts et al., 2019). To describe the *in vivo* pharmacokinetics and biodistribution of antibodies, standard two-compartment PK models were used and we were able to derive one consistent set of parameters that can simultaneously characterize the collected *in vivo* pharmacokinetics of multiple antibody drugs with good accuracy, and this set of PK parameter served as a starting point for further preclinical efficacy estimations. We also assumed that the concentration of antibodies within the tumor microenvironment can be calculated using standard partition coefficients and their corresponding plasma concentrations (Bordeau et al., 2022; Khaowroongrueng et al., 2021). For the modeling of T cell intracellular signaling (especially hub protein phosphorylation or activation, e.g., ERK, NFκB), we assumed mass action-based multiplicative signal transduction in formulating the reactions while the upstream signaling modulators were unconsumed during such reactions. For the modeling of tumor growth *in vitro* and *in vivo*, we implemented first-order growth kinetics with prespecified maximal tumor volume. The tumor cell clearance rate was driven by Hill-type promotion or inhibition calculated for different checkpoint complexes (e.g., level of bound PD-1/PDL1), activating state of T cells (e.g., level of activated hub proteins) and secreted cytokines (e.g., level of IFNγ). The binding rules for immune checkpoints as well as for antibody drugs with their targets were all mass action-based; for bispecific antibodies, they were allowed to first form dimers (drug-checkpoint#1 or drug-checkpoint#2) and then trimers (drug-checkpoint#1-checkpoint#2) as aforementioned. A detailed summary of model species, parameters, exemplar reactions, and their descriptions is provided in Supplementary Table S1.

After the T cell intracellular signaling module was calibrated by *in vitro* data, we then utilized a large dataset including *in vitro* cytotoxicity data as well as *in vivo* tumor growth inhibition data for the stepwise model calibration and validation. We selectively included experimental datasets of time-course tumor growth profiles (e.g., TGI under different drug treatment scenarios, including different doses and combinations) performed in the MC38 syngeneic mouse model as our primary source for model calibration and validation, while a small number of TGI data performed in other syngeneic mouse models were also considered during model formulation as secondary data sources to further enhance the general applicability of the model (see Supplementary Table S1 for details). During this translational calibration process (of model calibration from *in vitro* to *in vivo* levels), we kept the model structure (T-Tumor-APC) and reactions unchanged and added the *in vivo* mouse PK modules for antibody therapeutics; to better characterize the interplay and contribution of different checkpoints in modulating T cell activation and tumor killing, regulatory strength of immune checkpoints (km4∼km12) were re-optimized based on *in vivo* data (including time-course TGI data covering all checkpoint interventions), and tumor cell proliferation as well as death rates were also subjected to adjustment since different *in vivo* studies observed large variations in the tumor growth profiles of the control (untreated) mice. At the *in vivo* level, to better mimic the tumor microenvironment, we set the initial conditions of different cells within the tumor using literature knowledge (1e5 tumor cells, 370 T cells and 10 APCs per 1 mm^3^ of tumor) (Li et al., 2022; Del Monte, 2009; Fearnley et al., 1999). Subsequently during the model validation stage against *in vivo* data, we enforced that all parameters were kept unchanged (compared to the calibration stage) for each dataset and the model simulation results were validated respectively against unused experimental data, which primarily included *in vivo* TGI profiles obtained in the scenarios of new (e.g., higher) antibody treatment doses or new combinations.

In the subsequent analyses using virtual mouse populations, we systematically sampled a comprehensive set of model parameters within physiological ranges that included initial tumor volume, tumor growth and death rates, checkpoint expression levels, initial cell counts, etc. We typically considered 100 sets of distinct parameterizations as one virtual mouse population, with each parametrization representing one individual virtual mouse. The final model was implemented using the MATLAB Simbiology toolbox (MathWorks, Natick, MA) in terms of mass-action or Hill-type kinetic rate laws. Model simulations were executed using the ode15s solver. During model formulation, model parameters were globally optimized and estimated using the *patternsearch* function. A summary of model species, parameters, reactions, and their descriptions is provided in Supplementary Table S1.

### Global sensitivity analysis and statistical analysis

Global sensitivity analysis was conducted using the Sobol method under the *in vivo* no treatment scenario. By assuming a starting tumor volume of 100 mm^3^ *in vivo* and using the predicted tumor volume on day 25 as the output of interest, the most influential parameters were then identified and ranked (visualized in terms of total order sensitivity indices). The Wilcoxon rank-sum test was used to evaluate the model-predicted TGI profiles in response to antibody treatments at different dose levels in the virtual mouse population. Specifically, we compared the simulated TGI distribution of the lowest dose group with that of the higher dose groups, and P values <0.05 were considered statistically significant.

## Data Availability

The original contributions presented in the study are included in the article/Supplementary Material, further inquiries can be directed to the corresponding authors.
