# Supplementary material for: A mechanistic quantitative systems pharmacology model platform for translational efficacy evaluation and checkpoint combination design of bispecific immuno-modulatory antibodies
Source: Front Pharmacol. 2025 Apr 10;16:1571844. doi: 10.3389/fphar.2025.1571844 (PMC12018249; doi:10.3389/fphar.2025.1571844)
Supplement: Supplementary file 1 [file DataSheet1.pdf]

A mechanistic quantitative systems pharmacology model platform for translational efficacy evaluation and checkpoint combination design of bispecific immuno-modulatory antibodies

Yiyang Xu[1#], Siyuan Yang[1#], Qi Rao[1#], Yuan Gao[2], Guanyue Zhou[3], Dongmei Zhao[3], Xinsheng Shi[3], Yi Chai[4*], Chen Zhao[1,5*]

1 School of Pharmacy, Nanjing Medical University, Nanjing, China, 210000

2 QSPMed Technologies, Nanjing, China, 210000

3 Nanjing Sanhome Pharmaceutical Co., Ltd., Nanjing, China, 221116

4 Phase I Clinical Trial Unit, The First Affiliated Hospital of Nanjing Medical University, Nanjing, China, 210000

5 Department of Oncology, The First Affiliated Hospital of Nanjing Medical University, Nanjing, China, 210000

This file includes:

Supplemental Fig. S1. Model-based quantitative characterization of T cell intracellular signaling. See also Fig. 2.

Supplemental Fig. S2. Additional model calibration using in vitro data. See also Fig. 3.

Supplemental Fig. S3. Model-based quantitative characterization of time-course drug pharmacokinetics in mice. See also Fig. 3.

Supplemental Fig. S4. Additional model calibration using in vivo data on antibody-induced tumor growth inhibition – part 1 (BsAbs). See also Fig. 3.

Supplemental Fig. S5. Additional model calibration using in vivo data on antibody-induced tumor growth inhibition – part 2 (mAbs). See also Fig. 3.

Supplemental Fig. S6. Additional model validation using in vivo data on antibody-induced tumor growth inhibition. See also Fig. 4.

Supplemental Fig. S7. Antibody dosing regimen analyses and projection of combination efficacy. See also Fig. 6.

Supplemental Fig. S8. Model-based anti-tumor efficacy analyses of combining SHP2 inhibition with checkpoint regulation. See also Fig. 6.

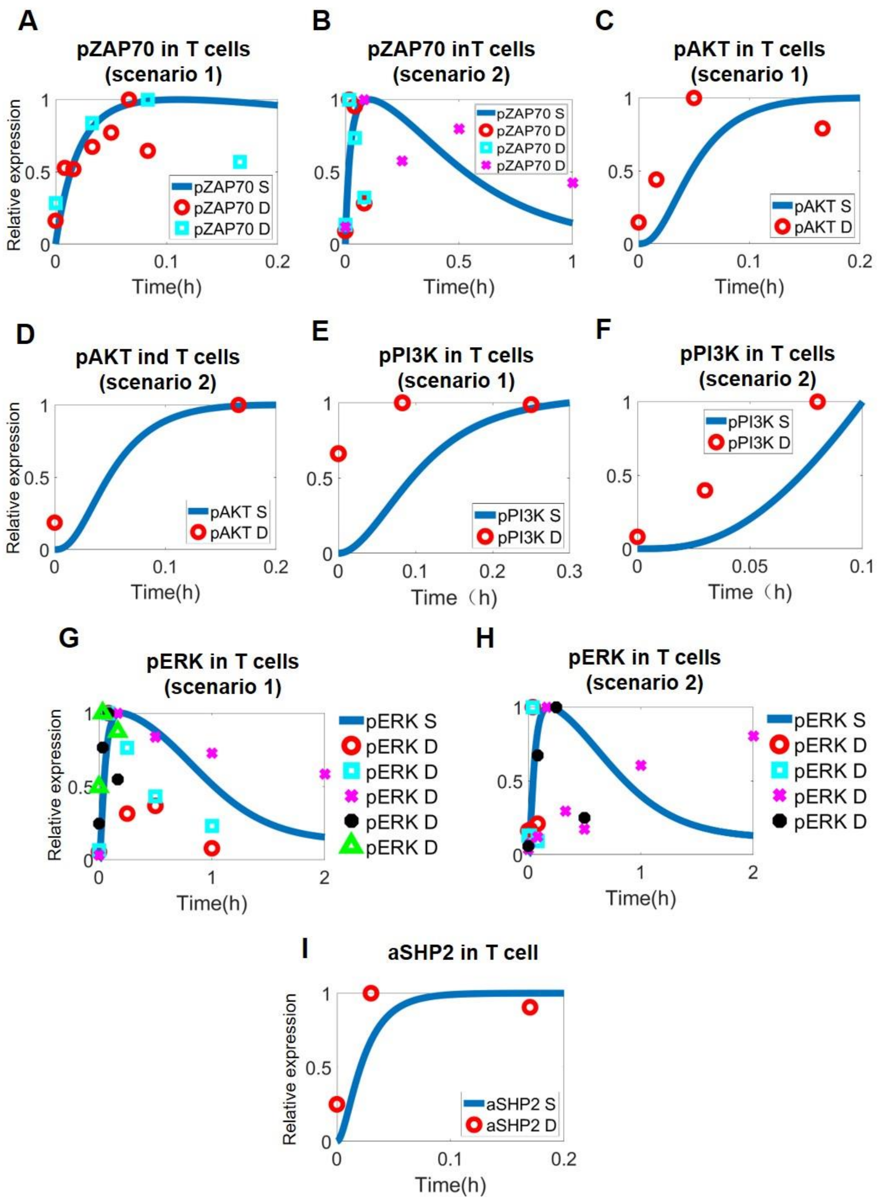

Figure S1. Model-based quantitative characterization of T cell intracellular signaling. Upon T-cell activation, ZAP70 was rapidly phosphorylated, as shown by model simulation and experimental data in scenarios of **(A)** anti-CD3 treatment (data from Williams et al.[1] and Kästle et al.[2]), and **(B)** anti-CD3 and anti-CD28 treatment (data from Kästle et al.[2] and Rodriguez-Peña et al. [3]). **(C)** Downstream activation of AKT by phosphorylation under anti-CD3 activation (data from Kassem et al.[4]). **(D)** Downstream activation of AKT by phosphorylation under anti-CD3 and antiCD28 activation (data from Wang et al.[5]). **(E)** Downstream activation of PI3K by phosphorylation under anti-CD3 and antiCD28 activation (data from Alcázar et al.[6]). **(F)** Downstream activation of PI3K by phosphorylation under T-B cell co-culture (data from Zhao et al.[7]). **(G)** Downstream activation of ERK by phosphorylation under anti-CD3 activation (data from Kästle et al.[2], Fujiwara et al.[8], Tewari et al.[9]). **(H)** Downstream activation of ERK by phosphorylation under anti-CD3 and antiCD28 activation (data from Rodriguez-Peña et al.[3], Wang et al.[5], Zheng et al.[10]). **(I)** Downstream activation of SHP2 under T-B cell co-culture (data from Marasco et al[11]). **(A-I)** Y axes are relative expression levels (normalized to their respective maximum values). S, simulation; D, experimental data.

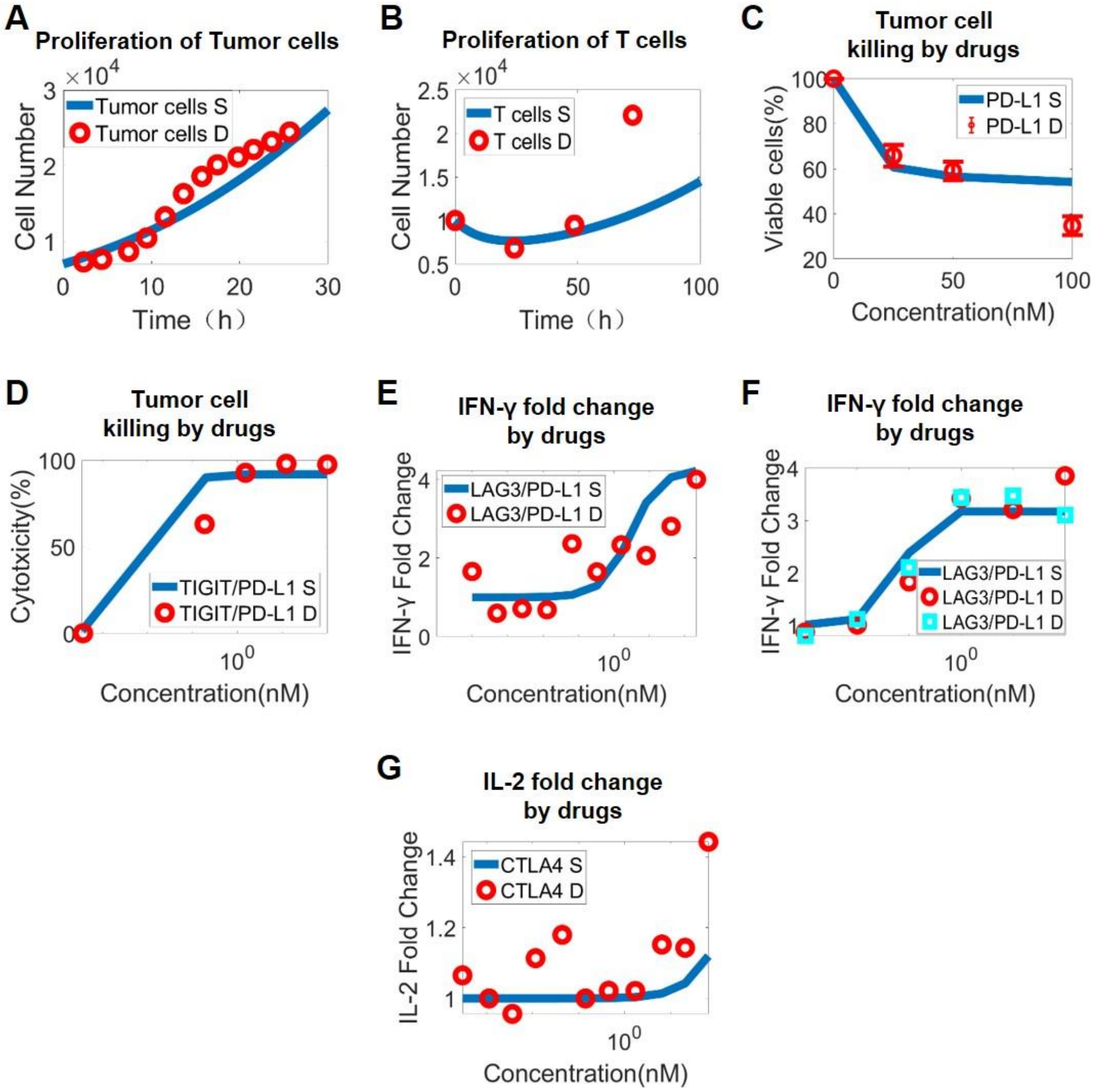

Figure S2. Additional model calibration using in vitro data. The QSP model can quantitatively capture **(A)** the growth of tumor cells over time(data from Muik et al.[12]) and **(B)** the time-dependent proliferation of T cells (data from Koenen et al.[13]). The integrated in vitro QSP model captures the dose response relationship of **(C)** anti-tumor cytotoxicity of anti-PD-L1 antibody (data from Passariello et al.[14]), **(D)** anti-tumor cytotoxicity of anti-TIGIT/PD-L1 bispecific antibody (data from Zhong et al.[15]), as well as increase in **(E-F)** IFN-γ release after anti-LAG3/PD-L1 bispecific antibody treatment (data from Jiang et al.[16], Kraman et al.[17]), and **(G)** IL-2 release after anti-CTLA4 antibody treatment (data from Li et al.[18]).

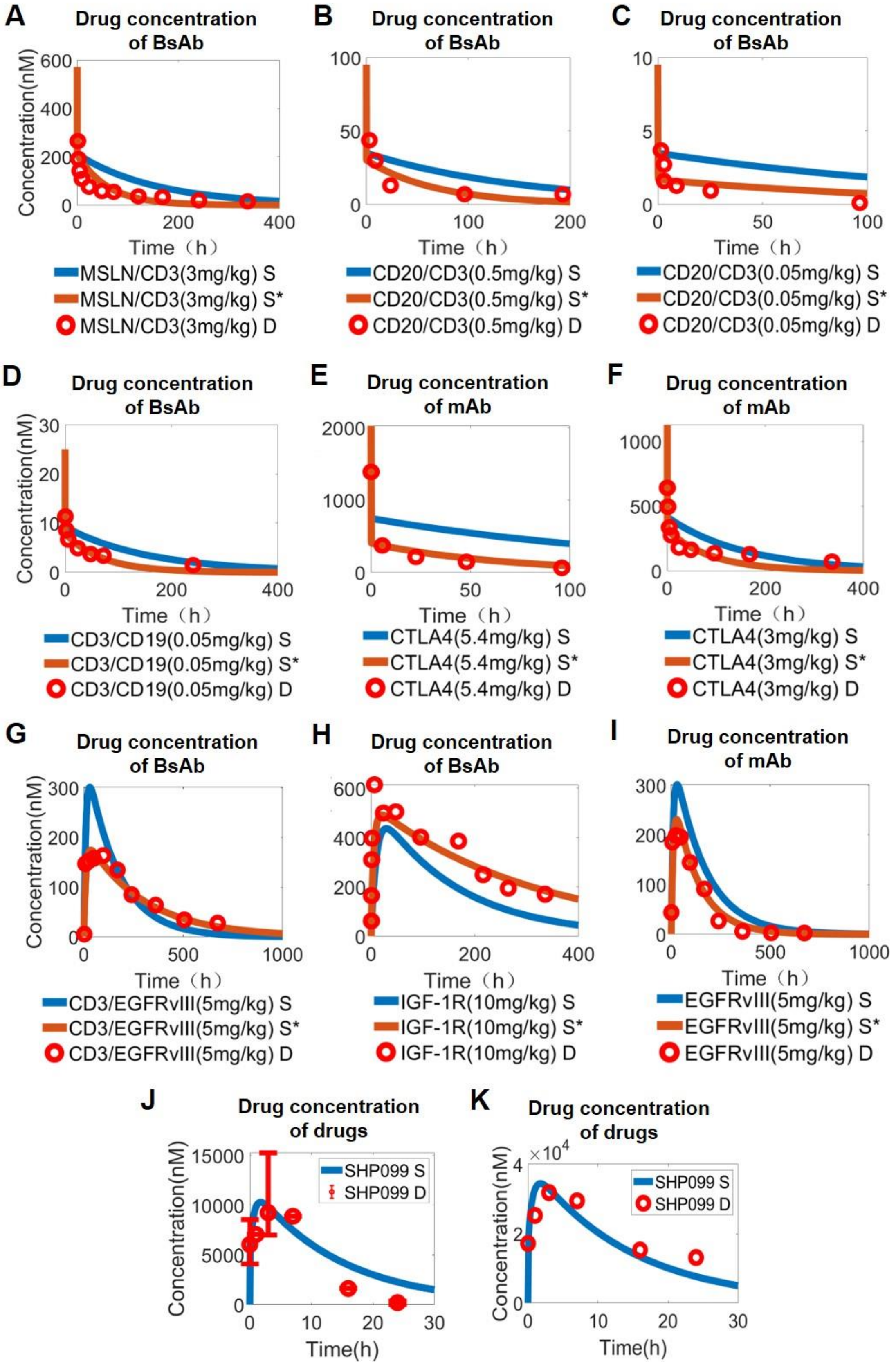

Figure S3. Model-based quantitative characterization of time-course drug pharmacokinetics in mice. The QSP model can quantitatively capture **(A)** plasma pharmacokinetics of anti-MSLN/CD3 bispecific antibody in mice (data from Yoon et al.[19]), **(B-C)** plasma

pharmacokinetics of anti-CD20/CD3 bispecific antibody in mice (data from Ferl et al.[20]), **(D)** plasma pharmacokinetics of anti-CD3/CD19 bispecific antibody in mice (data from Betts et al.[21]), **(E-F)** plasma pharmacokinetics of anti-CTLA4 antibody in mice (data from Gan et al.[22]), **(G)** plasma pharmacokinetics of anti-CD3/EGFRvIII bispecific antibody in mice (data from Sun et al.[23]), **(H)** plasma pharmacokinetics of anti-IGF-1R antibody in mice (data from Dong et al.[24]), **(I)** plasma pharmacokinetics of anti-EGFRvIII antibody in mice (data from Sun et al.[23]),and **(J-K)** plasma pharmacokinetics of SHP099 in mice (data from Garcia Fortanet et al.[25]). S, simulation; S*, optimized simulation after data-specific calibration; D, experimental data.

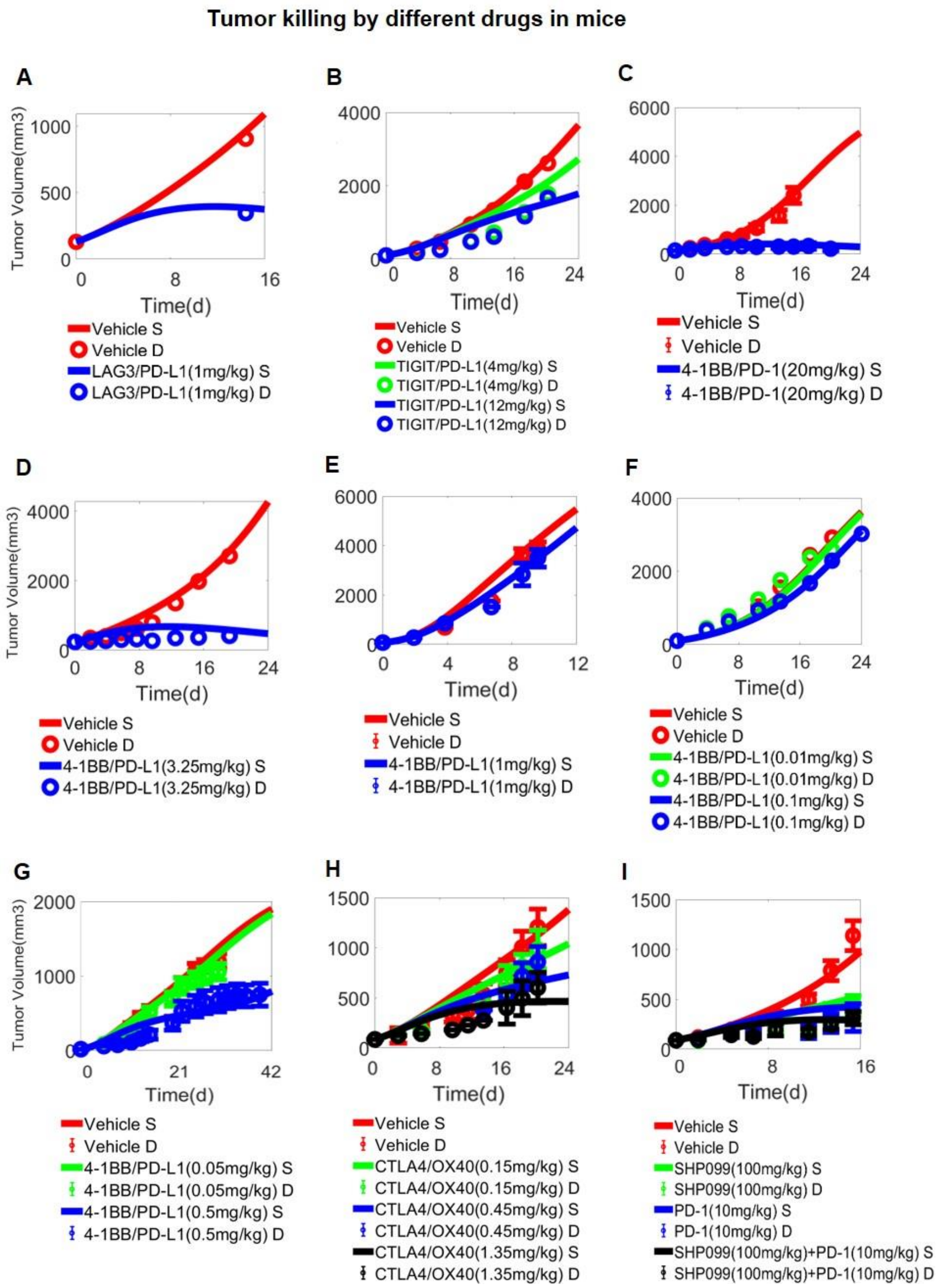

Figure S4. Additional model calibration using in vivo data on antibody-induced tumor growth inhibition – part 1 (BsAbs). In vivo antitumor activity of different antibody treatment regimens targeting immune checkpoints (and administered at different doses) as characterized by the QSP model; examples shown here include **(A)** anti-LAG3/PD-L1 bispecific antibody (data from Jiang et al.[16]) **(B)**anti-TIGIT/PD-L1 bispecific antibody (data from Zhong et al.[15]), **(C)** anti-4-1BB/PD-1 bispecific antibody (data from Qiao et al.[26]), **(D-G)** anti-4-1BB/PD-L1 bispecific antibody (data from Yuwen et al.[27], Peper-Gabriel et al.[28], Muik et al.[12]), **(H)** anti-CTLA4/OX40 bispecific antibody (data from Kvarnhammar et al.[29]), and **(I)** SHP099 and anti-PD-1 antibody(data from Wang et al.[30]). S, simulation; D, experimental data.

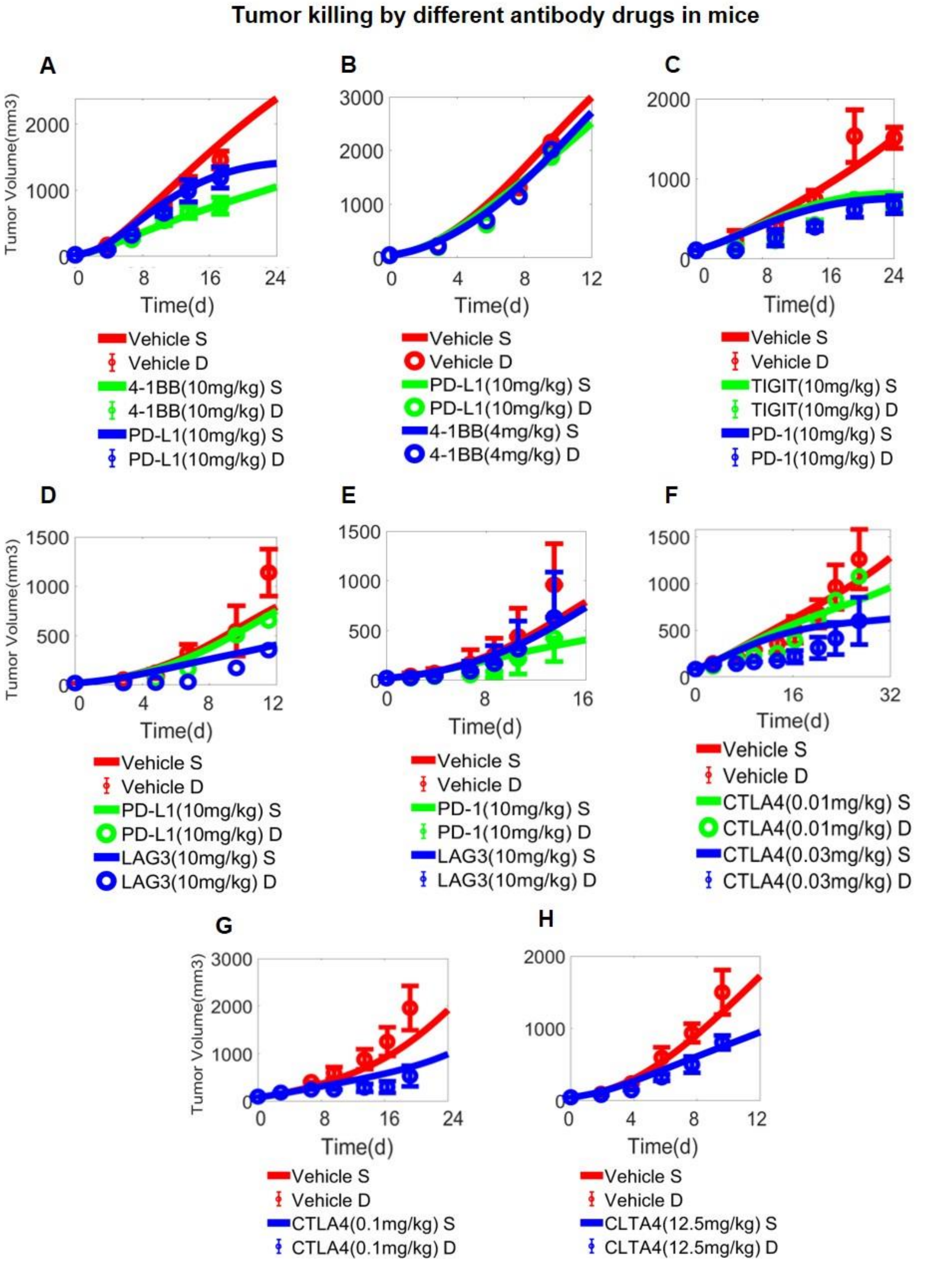

Figure S5. Additional model calibration using in vivo data on antibody-induced tumor growth inhibition – part 2 (mAbs). In vivo antitumor activity of different antibody treatment regimens targeting immune checkpoints (and administered at different doses) as characterized by the QSP model; examples shown here include **(A-B)** anti-4-1BB and anti-PD-L1 antibodies (data from Cheng et al.[31], Yuwen et al.[27]), **(C)** anti-TIGIT and anti-PD-1 antibodies (data from Shao et al.[32]), **(D-E)** anti-LAG3 and anti-PD-1 antibodies (data from Kraman et al.[17], Lecocq et al.[33]), **(F-H)** anti-CTLA4 antibody (data from Gan et al.[22], Du et al.[34]).S, simulation; D, experimental data.

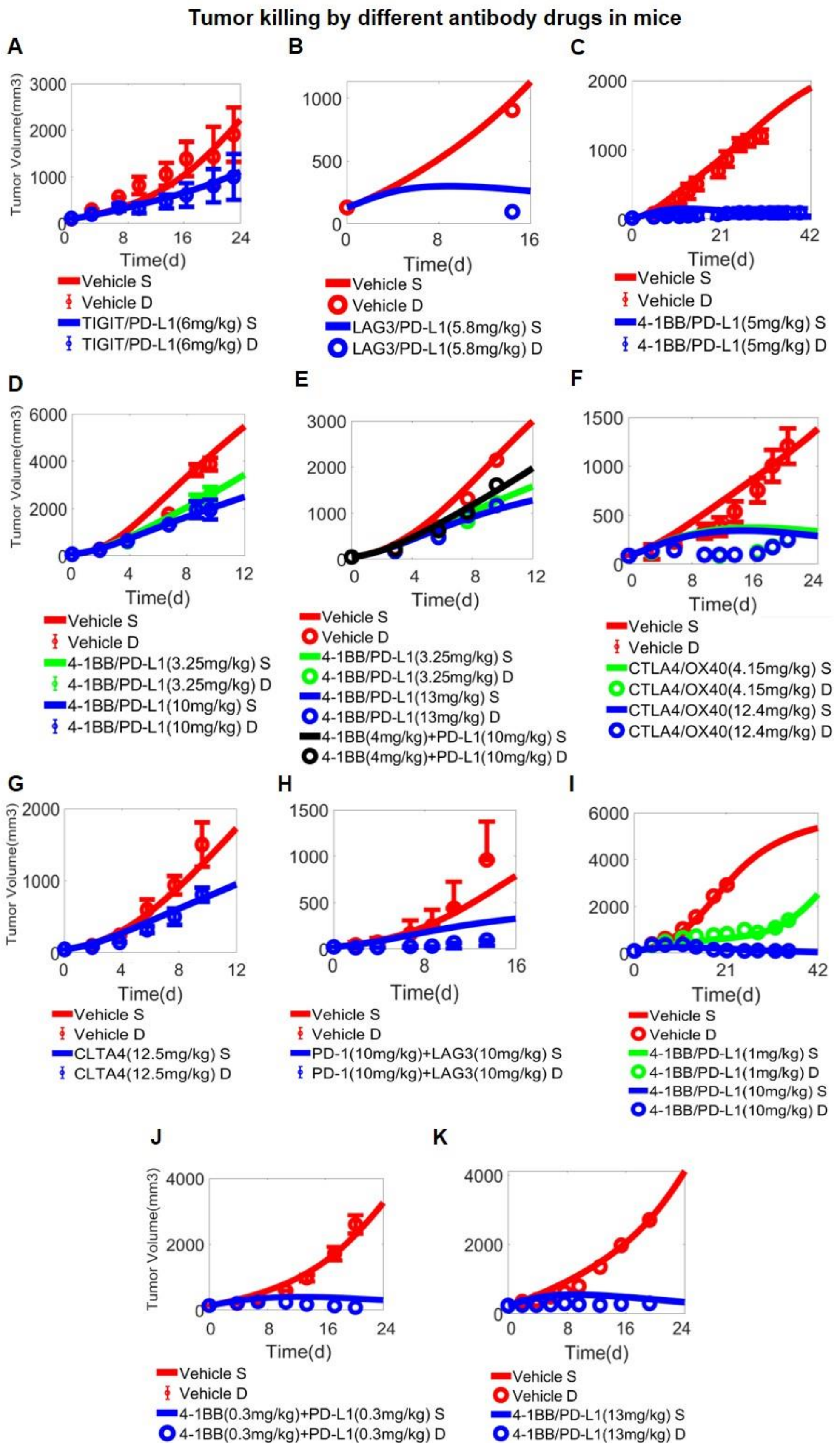

Figure S6. Additional model validation using in vivo data on antibody-induced tumor growth inhibition. In vivo antitumor activity of different antibody treatment regimens targeting immune checkpoints (and administered at different doses) as predicted by the QSP model; examples shown

here include **(A)** anti-TIGIT/PD-L1 bispecific antibody (data from Xiao et al.[35]), **(B)** anti-LAG3/PD-L1 bispecific antibody (data from Jiang et al.[16]), **(C-E)** anti-4-1BB/PD-L1 bispecific antibody (data from Muik et al.[12], Yuwen et al.[27]), **(F)** anti-CTLA4/OX40 bispecific antibody (data from Kvarnhammar et al.[36]), **(G)** anti-CTLA4 antibody (data from Du et al.[34]), **(H)** anti-LAG3 and anti-PD-1 antibodies (data from Lecocq et al.[33]), **(I)** anti-4-1BB/PD-L1 bispecific antibody (data from Peper-Gabriel et al.[28]), **(J)** anti-4-1BB and anti-PD-L1 antibodies (data from Cheng et al.[31]), **(K)** anti-4-1BB/PD-L1 bispecific antibody (data from Yuwen et al.[27]). S, simulation; D, experimental data.

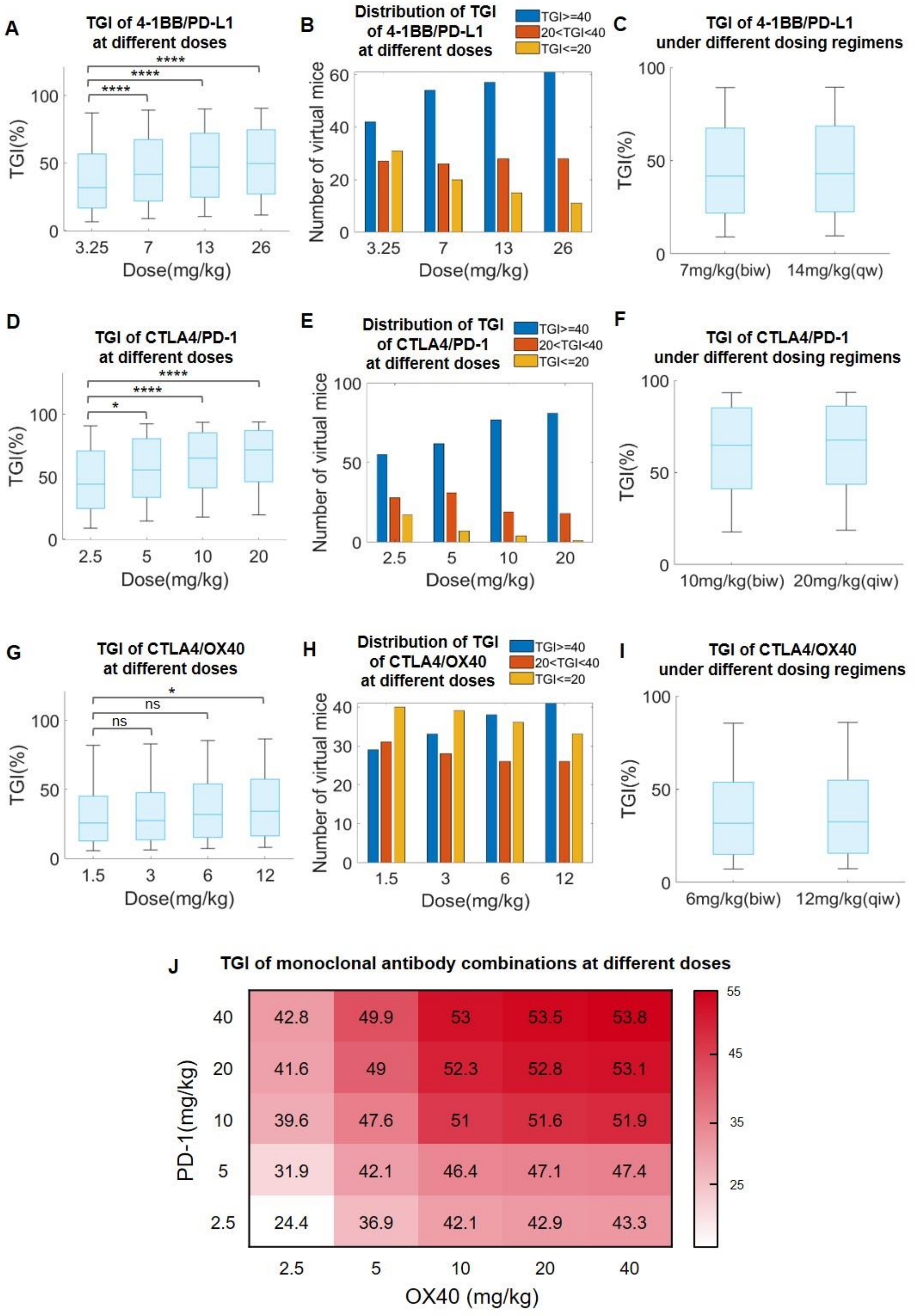

Figure S7. Antibody dosing regimen analyses and projection of combination efficacy. (A) Predicted TGI in the virtual mouse population after anti-4-1BB/PD-L1 BsAb treatment at doses of 3.25~26 mg/kg, and **(B)** distribution of TGI response depth (percentages of mice with TGI>=40%, 20%<TGI<40%, and TGI<=20%) at different doses. **(C)** Predicted TGI in response to anti-4-1BB/PD-L1 BsAb treatment at 7 mg/kg biw (twice per week) and 14 mg/kg qw (once per week)

dosing regimens. **(D)** Predicted TGI in the virtual mouse population after anti-CTLA4/PD-1 BsAb treatment at doses of 2.5~20 mg/kg, and **(E)** distribution of TGI response depth at different doses. **(F)** Predicted TGI in response to anti-CTLA4/PD-1 BsAb treatment at 10 mg/kg biw and 20 mg/kg qw regimens. **(G)** Predicted TGI in the virtual mouse population after anti-CTLA4/OX40 BsAb treatment at doses of 1.5~12 mg/kg, and **(H)** distribution of TGI response depth at different doses. **(I)** Predicted TGI in response to anti-CTLA4/OX40 BsAb treatment at 6 mg/kg biw and 12 mg/kg qw regimens. **(J)** Predicted population-level TGI heatmap for the combination of anti-PD-1 and anit-OX40 antibodies. nsP > 0.05,∗P < 0.05, ∗∗∗∗P <0.0001. Statistical analyses were performed using Wilcoxon rank-sum test.

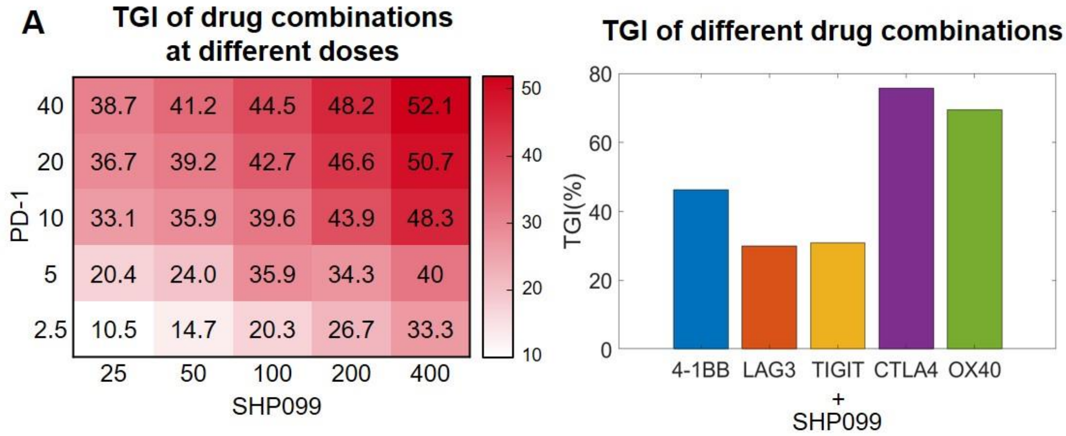

Figure S8. Model-based anti-tumor efficacy analyses of combining SHP2 inhibition with checkpoint regulation. (A) Predicted population-level TGI heatmap for the combination of anti-PD-1 and SHP099 (SHP2 inhibitor). **(B)** Predicted TGI for SHP099 combined with antibodies targeting other immune checkpoints.

References

1. Williams, B. L. *et al.* Phosphorylation of Tyr319 in ZAP-70 is required for T-cell antigen receptor-dependent phospholipase C-γ1 and Ras activation. *The EMBO Journal* **18**, 1832–1844 (1999).

2. Kästle, M. *et al.* Tyrosine 192 within the SH2 domain of the Src-protein tyrosine kinase p56Lck regulates T-cell activation independently of Lck/CD45 interactions. *Cell Communication and Signaling* **18**, 183 (2020).

3. Rodriguez-Peña, A. B. *et al.* Enhanced T-cell activation and differentiation in lymphocytes from transgenic mice expressing ubiquitination-resistant 2KR LAT molecules. *Gene Ther* **22**, 781–792 (2015).

4. Kassem, S. *et al.* A Natural Variant of the T Cell Receptor-Signaling Molecule Vav1 Reduces Both Effector T Cell Functions and Susceptibility to Neuroinflammation. *PLOS Genetics* **12**, e1006185 (2016).

5. Wang, X. *et al.* B7-H4 Treatment of T Cells Inhibits ERK, JNK, p38, and AKT Activation. *PLOS ONE* **7**, e28232 (2012).

6. Alcázar, I. *et al.* p85β phosphoinositide 3-kinase regulates CD28 coreceptor function. *Blood* **113**, 3198–3208 (2009).

7. Zhao, Y. *et al.* Antigen-Presenting Cell-Intrinsic PD-1 Neutralizes PD-L1 in cis to Attenuate PD-1 Signaling in T Cells. *Cell Rep* **24**, 379-390.e6 (2018).

8. Fujiwara, Y. *et al.* The GPR171 pathway suppresses T cell activation and limits antitumor immunity. *Nat Commun* **12**, 5857 (2021).

9. Tewari, R., Shayahati, B., Fan, Y. & Akimzhanov, A. M. T cell receptor–dependent S-acylation

of ZAP-70 controls activation of T cells. *Journal of Biological Chemistry* **296**, (2021).

10.Zheng, Y., Fang, Y.-C. & Li, J. PD-L1 expression levels on tumor cells affect their immunosuppressive activity. *Oncology Letters* **18**, 5399–5407 (2019).

11.Marasco, M. *et al.* Molecular mechanism of SHP2 activation by PD-1 stimulation. *Science Advances* **6**, eaay4458 (2020).

12.Muik, A. *et al.* An Fc-inert PD-L1×4-1BB bispecific antibody mediates potent anti-tumor immunity in mice by combining checkpoint inhibition and conditional 4-1BB co-stimulation. *Oncoimmunology* **11**, 2030135 (2022).

13.Koenen, P. *et al.* Mutually exclusive regulation of T cell survival by IL-7R and antigen receptor-induced signals. *Nat Commun* **4**, 1735 (2013).

14.Passariello, M. *et al.* Novel Human Anti-PD-L1 mAbs Inhibit Immune-Independent Tumor Cell Growth and PD-L1 Associated Intracellular Signalling. *Sci Rep* **9**, 13125 (2019).

15.Zhong, Z. *et al.* Development of a bispecific antibody targeting PD-L1 and TIGIT with optimal cytotoxicity. *Sci Rep* **12**, 18011 (2022).

16.Jiang, H. *et al.* PD-L1/LAG-3 bispecific antibody enhances tumor-specific immunity. *Oncoimmunology* **10**, 1943180 (2021).

17.Kraman, M. *et al.* FS118, a Bispecific Antibody Targeting LAG-3 and PD-L1, Enhances T-Cell Activation Resulting in Potent Antitumor Activity. *Clin Cancer Res* **26**, 3333–3344 (2020).

18.Li, Y. *et al.* Discovery and preclinical characterization of the antagonist anti-PD-L1 monoclonal antibody LY3300054. *J Immunother Cancer* **6**, 31 (2018).

19.Yoon, A. *et al.* A Novel T Cell-Engaging Bispecific Antibody for Treating Mesothelin-Positive Solid Tumors. *Biomolecules* **10**, 399 (2020).

20. Ferl, G. Z. *et al.* A Preclinical Population Pharmacokinetic Model for Anti-CD20/CD3 T-Cell-Dependent Bispecific Antibodies. *Clinical and Translational Science* **11**, 296–304 (2018).

21. Betts, A. *et al.* A Translational Quantitative Systems Pharmacology Model for CD3 Bispecific Molecules: Application to Quantify T Cell-Mediated Tumor Cell Killing by P-Cadherin LP DART®. *AAPS J* **21**, 66 (2019).

22. Gan, X. *et al.* An anti-CTLA-4 heavy chain-only antibody with enhanced Treg depletion shows excellent preclinical efficacy and safety profile. *Proc Natl Acad Sci U S A* **119**, e2200879119 (2022).

23. Sun, R. *et al.* A Rational Designed Novel Bispecific Antibody for the Treatment of GBM. *Biomedicines* **9**, 640 (2021).

24. Dong, J. *et al.* Stable IgG-like bispecific antibodies directed toward the type I insulin-like growth factor receptor demonstrate enhanced ligand blockade and anti-tumor activity. *J Biol Chem* **286**, 4703–4717 (2011).

25. Garcia Fortanet, J. *et al.* Allosteric Inhibition of SHP2: Identification of a Potent, Selective, and Orally Efficacious Phosphatase Inhibitor. *J. Med. Chem.* **59**, 7773–7782 (2016).

26. Qiao, Y. *et al.* Cancer immune therapy with PD-1-dependent CD137 co-stimulation provides localized tumour killing without systemic toxicity. *Nat Commun* **12**, 6360 (2021).

27. Yuwen, H. *et al.* ATG-101, a tetravalent PD-L1×4-1BB bispecific antibody, augments anti-tumor immunity through PD-L1 blockade and PD-L1-directed 4-1BB activatio. Preprint at https://doi.org/10.21203/rs.3.rs-2146670/v1 (2022).

28. Peper-Gabriel, J. K. *et al.* The PD-L1/4-1BB Bispecific Antibody–Anticalin Fusion Protein PRS-344/S095012 Elicits Strong T-Cell Stimulation in a Tumor-Localized Manner. *Clinical Cancer*

Research **28**, 3387–3399 (2022).

29. Kvarnhammar, A. M. *et al.* The CTLA-4 x OX40 bispecific antibody ATOR-1015 induces anti-tumor effects through tumor-directed immune activation. *Journal for ImmunoTherapy of Cancer* **7**, 103 (2019).

30. Wang, Y. *et al.* SHP2 blockade enhances anti-tumor immunity via tumor cell intrinsic and extrinsic mechanisms. *Sci Rep* **11**, 1399 (2021).

31. Cheng, L. *et al.* A humanized 4-1BB-targeting agonistic antibody exerts potent antitumor activity in colorectal cancer without systemic toxicity. *Journal of Translational Medicine* **20**, 415 (2022).

32. Shao, Q. *et al.* TIGIT Induces (CD3+) T Cell Dysfunction in Colorectal Cancer by Inhibiting Glucose Metabolism. *Frontiers in Immunology* **12**, (2021).

33. Lecocq, Q. *et al.* Nanobody nuclear imaging allows noninvasive quantification of LAG-3 expression by tumor-infiltrating leukocytes and predicts response of immune checkpoint blockade. *Journal of Nuclear Medicine* (2021) doi:10.2967/jnumed.120.258871.

34. Du, X. *et al.* A reappraisal of CTLA-4 checkpoint blockade in cancer immunotherapy. *Cell Res* **28**, 416–432 (2018).

35. Xiao, Y. *et al.* Discovery of a novel anti PD-L1 X TIGIT bispecific antibody for the treatment of solid tumors. *Cancer Treat Res Commun* **29**, 100467 (2021).

36. Kvarnhammar, A. M. *et al.* The CTLA-4 x OX40 bispecific antibody ATOR-1015 induces anti-tumor effects through tumor-directed immune activation. *J Immunother Cancer* **7**, 103 (2019).
